# *In vitro* screening of compounds for targeting gastric cancer with Y220C p53 mutation: a molecule combining zinc chelation and a Michael acceptor drives *CDKN1* and *BBC3* expression to restore a p53-dependent cytotoxicity

**DOI:** 10.1080/14756366.2026.2638836

**Published:** 2026-05-21

**Authors:** Simon Nannini, Céline Sieffert, Andrew McGown, Xin-Yue Gao, Amanda Jarvis, Georges E. Kostakis, Antal Galvacsi, Csilla Kallay, Ruxandra Moraru, Matthias G. Baud, Sebastian Mandel, Dimitros-Ilias Balourdas, Andreas C. Joerger, Christophe Orvain, Simon Peschard, Audrey Nion, Georg Mellitzer, Sophie Lottiaux, John Spencer, Isabelle Gross, Christian Gaiddon

**Affiliations:** ^a^ “Streinth” Laboratory, University of Strasbourg, INSERM UMR_S 1113, Strasbourg, France; ^b^ “SMART” Laboratory, University of Strasbourg, INSERM UMR_S 1113, Strasbourg, France; ^c^ Sussex Drug Discovery Centre, School of Life Sciences, University of Sussex, Brighton, East Sussex, UK; ^d^ EastCHEM School of Chemistry, University of Edinburgh, Joseph Black Building, Edinburgh, UK; ^e^ Department of Chemistry, School of Life Sciences, University of Sussex, Brighton, East Sussex, UK; ^f^ Department of Inorganic and Analytical Chemistry, University of Debrecen, Debrecen, Hungary; ^g^ School of Chemistry, University of Southampton, Southampton, UK; ^h^ Institute of Pharmaceutical Chemistry, Goethe University, Frankfurt am Main, Germany; ^i^ Buchmann Institute for Molecular Life Sciences and Structural Genomics Consortium (SGC), Frankfurt am Main, Germany; ^j^ "HERIT" Laboratory, University of Strasbourg, INSERM UMR 1260, Strasbourg, France; ^k^ “Streinth” Laboratory, University of Strasbourg, CNRS UMR7242, Illkirch, France

**Keywords:** p53, covalent drugs, targeted therapy, gastric cancer, metal chelation

## Abstract

Point mutations in p53 favour tumour aggressivity, particularly in gastric cancer (GC), and offer a target for small molecule-based anticancer treatments. This study focused on the p53-Y220C mutation, which causes p53 misfolding due to thermal instability associated with the creation of a pocket that may accommodate small molecules. This mutation also creates an additional free cysteine thiol group that may react with Michael acceptors. Using an integrated *in silico* and *in vitro* approach, four compounds (AG1, AG2, AG3, and RK349) were screened for potential reactivation of p53-Y220C in GC cells. AG3, a compound with zinc chelation and Michael acceptor properties, was found to induce p53 target gene expression via p53-dependent and -independent pathways. AG3 limited reactive oxygen species production, reducing toxicity to healthy cells. Furthermore, AG3 induced p53-dependent cytotoxicity and enhanced chemotherapy response. This study presents a novel compound with p53-Y220C reactivation potential, highlighting its promise for further development.

## GRAPHICAL ABSTRACT

**Figure:**
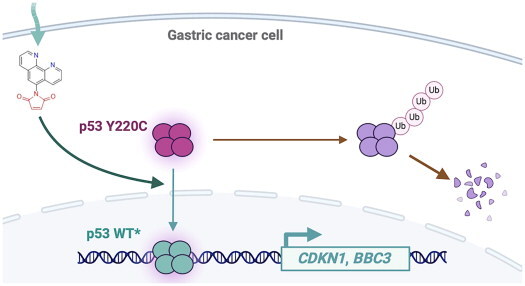

## Introduction

The transcription factor p53 is critical for cellular homeostasis. Implicated in numerous and often redundant pathways, p53 opposes tumorigenesis through anti-proliferative and pro-apoptotic activities^1^. These essential functions explain why over 50% of solid tumours are associated with mutations of p53. Most of these (∼70%) occur as missense mutations and are predominantly (95%) located in the central DNA-binding domain (DBD, 102–292)^2–5^. The consequences of these mutations can be divided into two groups: (i) mutations that eliminate direct DNA contacts (“contact” mutants) without altering the overall conformation of the protein, and (ii) mutations that perturb the protein structure (“structural” mutants), resulting in mis/unfolding, followed by rapid aggregation^6^. Some of those mutants are also associated with *de novo* pro-oncogenic features via co-aggregation with proteins that can inhibit anti-tumoral activity (e.g., co-aggregation with p63 and p73)^7^^,^^8^ or promote increased pro-tumoral activity (e.g., co-aggregation with YAP or NRF2)^9^^,^^10^. Tumours that display these mutations have a poorer prognosis, showing higher resistance to chemotherapy, more metastases, reduced anticancer immune landscape and lower survival rate compared to wild type (WT) p53 tumours. This is particularly true for gastric cancers (GC) in which mutations of p53 are considered as oncogenic drivers as they are detected at early stages of tumorigenesis and in up to 70% of tumors^11^. Platinum salts, such as oxaliplatin, are still standard treatment for GC and are known to cause DNA damage that induces p53 activity^12^. However, GC is associated with more than 75% resistance to chemotherapy and has a very poor outcome. It is currently the fourth deadliest cancer globally and is directly responsible for 100 000 deaths per year worldwide^11^.

Hence, strategies have been developed to either induce p53-independent cytotoxicity (e.g., innovative metal-based compounds)^13^^,^^14^ or to reactivate the p53 pathway using various means (peptides, small molecules, viruses expressing WT p53)^15–17^. Amongst those strategies, targeting p53 mutations is a cornerstone to improve clinical outcomes of patients diagnosed with cancer. As those mutations are only present in cancer cells, they represent ideal targets for strategies aimed at reducing the side effects of conventional chemotherapies (e.g., oxaliplatin) that are widely used^18^^,^^19^. Over the last two decades, several compounds that target mutations of p53 and restore its wild-type function inside tumour cells have been designed using various approaches^16^. For example, APR-246 has been shown to covalently modify several cysteine residues in the p53 DBD and help to maintain the functional folding of p53^20^^,^^21^. Its impact on cancer xenograft cells harbouring R175H and R273H mutations led to phase IB and II clinical trials for myelodysplastic disease^22^ and eventually an FDA approval for this disease. However, APR-246 is a promiscuous alkylator and also acts via off-target pathways (e.g., thiol depletion)^23^, which explains the effects in cells with DNA-contact mutants. Another way to improve the stability and folding of p53 is to target binding of the zinc ion by the DBD. For instance, ZMC1 is a metallochaperone molecule that improves the stability of p53 by increasing cellular zinc levels, but at the cost of significant off-target effects^24^. More recently, we have conceived bifunctional ligands L^I^ and L^H^ to reduce the misfolding of p53 through zinc binding with more specificity and fewer off-target effects^15^. Other studies have shown that many, but not all, structural mutants can be reactivated with arsenic trioxide, which stabilises the p53 DBD by coordinating three cysteines in the loop-sheet-helix motif^25^. Similar effects were also seen with antimony-based compounds, which target the same site but reactivate fewer mutants than arsenic trioxide^26^.

Overall, numerous approaches exist for targeting structural p53 cancer mutants. Despite their efficacy *in vitro*, many of these molecules are non-specific and show significant side effects. Indeed, many of these therapies have off-target effects, namely through reactive oxygen species (ROS) production, which have hampered their development in clinical trials.

The Y220C mutant is a key target in the field of p53 mutant reactivation research. It is the 9^th^ most frequent p53 cancer mutant, accounting for more than 100 000 new cancers each year worldwide^27^^,^^28^. This mutation is also one of the most frequent mutations found in GC and is associated with Approximately 1% of all tumours globally. The mutation of a tyrosine to a cysteine leads to an unstable DBD and creates a unique druggable crevice on the surface of the protein^29^, allowing the design of low molecular weight compounds that specifically target this mutant^30–32^. Importantly, this mutation also results in a free cysteine thiol (-SH) group that can potentially react with a Michael acceptor. What makes this mutant a particularly attractive target compared to other mutants such as R175H or DNA-contact mutants is its associated temperature-sensitive phenotype^16^. The Y220C mutant is therefore an excellent target for developing molecules that either directly or indirectly stabilise the protein, thereby restoring p53 signalling pathways in cells.

Herein, we selected, characterised, and compared four molecules (i.e., AG1, AG2, AG3, and RK349) that combine a Michael acceptor with a potential zinc-chelating moiety to evaluate their ability to restore a p53-dependent activity and cytotoxicity in cancer cells with a homozygous p53 Y220C mutation^33^. Due to its high lethality and limited therapeutic options, we focused our study primarily on gastric cancer and evaluated the cytotoxicity of these four molecules in several GC cell lines, but also other types of digestive cancer. We assessed their functional impact on p53 but also their potential off-target effects through induction of ROS and also tested the effects in non-tumour intestinal organoids. In addition, we benchmarked the four new molecules against the putative p53-reactivating compounds APR-246, ZMC1 and Phikan083.

## Materials and methods

### Cell lines

AGS (ATCC©, CRL-1739) and NUGC3 (JCRB0822**;** JCRB cell bank, Japan) cell lines are derived from human gastric adenocarcinoma expressing wild type and Y220C mutant p53, respectively. HCT116 (ATCC©, CCL247) and SW480 (ATCC©, CCL228) cell lines are derived from colon cancer expressing wild-type and R273H mutant p53, respectively. BxPC3 cells (ATCC©, CRL1687) are from pancreatic cancer expressing Y220C mutant p53. They were cultured in Roswell Park Memorial Institute medium 1640 (RPMI, Gibco) supplemented with 10% foetal calf serum (FCS, Gibco). The KMST-6 cell line (CVCL_2998), obtained from Riken, was established by immortalisation of human skin fibroblasts and cultured in Minimum Essential Medium (MEM, Gibco) supplemented with 10% FCS and 1% glutamine (Gibco).

### Chemical compounds

APR-246, ZMC1, PhiKan083 and Nutlin-3 were purchased from TOCRIS^®^. Oxaliplatin was purchased from Sigma Aldrich^®^ at 99% purity. AG1, AG2, AG3 and RK349 were synthesised according to literature procedures^33–36^ (for full details and characterisation see ESI). The compounds were prepared in a stock solution of dimethyl sulfoxide (grade for cell biology) at a 50 mM concentration (DMSO, VWR Chemicals). ZVAD-FMK and ferrostatin-1 were purchased from TEBU BIO and Euromedex France, respectively.

### MTT assay

MTT survival tests were performed with cells seeded in 96-well plates (AGS, HCT116, SW480, BxPC3: 10 000 cells/well; NUGC3: 20 000 cells/well; KMST-6: 30 000 cells/well) for 24 h and treated for 48 h (5 wells/condition). The supernatant was then aspirated and replaced by medium containing 0.5 mg/mL MTT tetrazolium salt (Thiazolyl Blue tetrazolium bromide, Thermo Scientific Chemicals) for 1.5 h (AGS, HCT116, SW480, BxPC3) or 2.5 h (NUGC3, KMST-6) before solubilisation of the tetrazolium salts with pure DMSO and measurement of the photon absorption by a Tristar2 Multimode Reader LB942 spectrometer (Berthold Technologies) at 590 nm. The differences in absorbance were normalised to the untreated control conditions. Statistical calculations and representations were performed on Prism V8.4 (GraphPad Software). The IC values were obtained by non-linear regression for the IC_50_ (50% of the theoretical maximum effect) and by plotting for the IC_75_ (75% of the theoretical maximum effect).

### Clonogenic assay

500 000 cells were seeded in 6-well plates. After 24 h, cells were treated with the compounds for 48 h (3 wells/condition). Next, cells were counted and seeded in another well (1000 cells/well) for 5 d. Colonies were counted after staining with crystal violet (0.1% for 10 min).

### ROS detection by DHE probe

200 000 cells were seeded on degreased glass coverslips placed in 12-well plates. After 24 h, they were treated for 4 or 18 h with the compounds. Incubation with 25 µM hydroethidine [dihydroethidium] (DHE, AAT Bioquest) and 3 μM of Hoechst 33342 (Sigma) in 10% FCS-PBS was performed for 30 min in the dark, followed by 3 PBS wash and 20 min of fixation with 1% formaldehyde (PFA, Citifluor-EMS). After washing, coverslips were mounted on slides with FluorSave Reagent (Millipore) for 1 h and stored at 4 °C. The fluorescence signal was captured for an excitation/emission spectrum of 460/490 nm for the Hoechst probe and 480/576 nm for the DHE probe. The fluorescent cell count was performed using Image J software by calculating the percentage of positive cells on three different images for each slide.

### RNA extraction and RT-qPCR

Cells were seeded in 6-well plates (500 000 cells/well) for 24 h and treated for 4–36 h with the compounds. Total RNA was extracted from both adherent cells and floating cells using Tri-reagent (MRC). Reverse transcription (RT) was performed with 4 µg of RNA using the High-Capacity RT kit without RNAsin (Applied Biosystems) as recommended. qPCR reactions were done in triplicate on a LightCycler^®^ 480 apparatus (Roche) using 1/100^th^ of RT, the LightCycler^®^ 480 SYBR Green Master mix (Roche) and specific oligonucleotide primers (Invitrogen, Table S1). Relative mRNA expression was calculated using the ΔΔCt method and *RPLPO*/*GAPDH* as reference genes. Data processing was performed on LightCycler 480^®^ software. Statistical analyses between conditions were performed by Mann-Whitney tests.

### Western blot

Cells were seeded in 6-well plates (500 000 cells**/**well) and treated for 4–24 h with the compounds. Adherent cells and floating cells were lysed with a NP40 (Igepal, Sigma, 1%) buffer and sonication. 30 µg of proteins were separated by SDS-PAGE (10%) and transferred to nitrocellulose membranes (Amersham Protran 0.45 μm, GE Healthcare). Membranes were saturated with 5% bovine serum albumin (BSA, Euromedex) in 0.1% Tween-Tris Buffered Saline for 45 min at room temperature (RT). Antibodies were diluted in the same buffer containing 1% BSA. Primary antibodies (O/N at 4 °C) included mouse monoclonal anti-p53 (DO-1, Santa Cruz Biotechnology; 1/1000), rabbit monoclonal anti-NRF2 (clone D1Z9C, Cell Signalling Technology; 1/1000), mouse monoclonal anti-actin (clone C4, Chemicon; 1/15 000). Secondary antibodies (45 min at RT) were either anti-rabbit or -anti-mouse IgG, HRP-linked (Cell Signalling Technology, 1/10 000). HRP activity was revealed with Immobilon Western Chemiluminescent HRP Substrate (Millipore), detected with a Pxie Imager (Syngene) and quantified with Fiji software.

### RNA interference

RNA interference for p53 was performed with cells seeded in 6-well plates (300 000 cells/well). The next day, synthetic duplexes of siRNA (siCtrl: Control siRNA duplex negative control; sip53: GGA AAC UAC UUC CUG AAA A; Eurogentec; 30 nM) were transfected with Lipofectamine RNAiMAX (Invitrogen) as recommended. After 24 h, the medium was removed and the cells were treated with the different compounds for 24 – 48 h.

### Organoids

Organoids were generated from either murine small intestine from intestinal crypts isolated from a 2-month old female C57BL/6 mouse (in-house stabulation, animal ethic committee number: Apafis N°16115) as previously described^15^. Mice experiments were performed in compliance with French administrative and ethical regulation (Apafis N°16115). After cervical dislocation of 3 months-old mice, the ileum was removed, rinsed with cold PBS (D. Dutscher), opened longitudinally, scraped with a glass slide to remove the villi and cut into 5 mm-fragments. These were vigorously washed in successive baths of antibiotics (Penicillin-Streptomycin 1% v/v, Gentamycin 0.2% v/v in PBS, Gibco). Next, the fragments were incubated for 30 min at 4 °C in EDTA (2 mM, Invitrogen) under vigorous agitation to detach the intestinal crypts from the underlying tissue. A filtration step (70 µm, Falcon) was performed to remove the villi before centrifugation (5 min, 260 g, 4 °C). The pelleted crypts were resuspended in 500 µL of cold PBS and counted. 200–400 crypts were homogenised in 20 µL of cold Matrigel (Basement Membrane Matrix GFR Phenol Red-free, Corning) and the suspension was deposited (“beaded”) into a well of a 48-well plate (CELLSTAR, Greiner Bio-one) preheated to 37 °C. The polymerised domes (< 10 min, 37 °C) were covered with 250 µL of Intesticult (Mouse Organoid Growth Medium, STEMCELL Tech.) containing antibiotics (Penicillin-Streptomycin 1% v/v, Fungizone 1% v/v, Gentamycin 0.08% v/v, Gibco) and an apoptosis inhibitor (Y-27632 10 µM, STEMCELL Tech.). Cysts were detected after 24–72 h and gradually developed buds as they increased in size. The medium was changed every 2–3 days and organoids split every 7–10 days. For mouse organoids and bright-field microscope follow-up, dissociated organoids were grown in 48-well plates (Greiner Bio-One) for 4 d and treated or not (NT) for 2 d with DMSO (Sigma-Aldrich, CT), AG1 (15 µM), AG3 (18 µM), PhiKan083 (37 µM), oxaliplatin 12 µM diluted in DMSO in triplicate. These concentrations correspond to the IC_50_ of each compound in NUGC3 cells. For human organoids, human ileal tissue, provided by Strasbourg University Hospitals, in compliance with all administrative and ethical regulations (HUS Ethic committee #CE-2021–142), was dissected on ice to isolate the mucosal layer. After gentamicin (50 ng/mL) and amphotericin (2.5 ng/mL) incubation, crypts were isolated as described^37^. Briefly, intestinal mucosa was sliced into 0.5 mm^3^ fragments, incubated for 30 min in sterile PBS with 2 mM EDTA on ice and vigorously shaken for 2 min. After filtration of the supernatant (100 µm cell strainer, Falcon), crypts were counted, concentrated by centrifugation (1200 rpm at 4 °C for 5 min) and resuspended into growth factor reduced Matrigel (Corning) at a density of 300 crypts per 20 µL Matrigel dome. Organoids were then cultivated for 7–10 d into Intesticult human Organoid Growth Medium (StemCell Tech.) supplemented with 10 µM of Rock-inhibitor Y27632 (StemCell Tech.) before passaging by enzymatic dissociation (TrypLE, Gibco; 10 min at 37 °C). Y27632 was added to the culture medium only for the first passage, then only for the first 2 d of culture at each passage. Organoids at passage 4 were treated 7 d after seeding in 8-well Lab-Tek^™^ II Chamber Slide (Nunc) with DMSO or AG3 (18 µM) for 24 or 48 h before PFA fixation and staining.

For immunofluorescence microscopy, mouse or human organoids were grown on 8-well LAB-TEK Chamber Slides (Nunc). After 48 h, organoids were fixed for 30 min with paraformaldehyde (EMS, 4% in 60 mM PIPES, 25 mM HEPES, 10 mM EGTA, 2 mM magnesium acetate) and permeabilized for 30 min in 0.5% Triton X-100 (Euromedex) at room temperature. After a 2 h-blocking at 37 °C in 5% BSA (Euromedex), cell nuclei were stained with DAPI (4′,6-diamidino-2-phenylindole, Sigma; 0.5 µg/mL) and actin was labelled by Phalloidin-TRITC (Sigma, 1:1000). Slides were mounted in ProLong Gold Antifade Mountant (Invitrogen) and analysed with an Axio Imager M2 microscope (Zeiss) coupled to a Hamamatsu’s camera Orca Flash 4v3 using the ApoTome.2 function (Zeiss) for optical sectioning.

### Additional materials and methods

Additional materials and methods are described in the Supporting Information. This includes primer and RNA sequences, protein expression and purification details, analytical methods; mass spectrometry, DSF, GSH off-target analyses. For chemistry, NMR and synthetic details, scanned spectra as well as stability studies are detailed.

### Statistics

Normality of the data distribution was checked by Shapiro-Wilk or Agostino & Pearson tests, and equality of variances was evaluated with the Brown-Forsythe test for all data. If these assumptions were met, statistical differences were determined using one-way ANOVA with Tukey’s *post hoc* test; otherwise, the non-parametric Kruskal-Wallis test with Dunn’s *post hoc* test was applied. Statistical analyses were performed using GraphPad Prism V8.4 software (GraphPad Software). For all analyses, significance was indicated as follows: *, *p* < 0.05; **, *p* < 0.01; *** *p* < 0.001.

## Results

### Transcriptional activity of wild-type p53 in gastric cancer cells

To monitor in the most relevant manner how our compounds may affect p53 in GC, our first objective was to select a set of p53 target genes to be used as markers of p53 transcriptional activity. To do so, we developed an original approach combining several sets of transcriptomics data: (i) our own data with the GC cell line AGS expressing wild-type p53 in which we silenced p53 using siRNA and/or activated p53 with oxaliplatin (Fig. S1a), (ii) published data from gastric tumours expressing wild-type or mutated p53 (TCGA database and (22)), (iii) published data from a GC cell line, LMSU, expressing a mutant p53 that has been silenced or not using shRNA^38^. Oxaliplatin was chosen because it is the gold standard for GC treatment and is known to cause DNA damage that induces p53 activity^12^.

**Figure 1.**
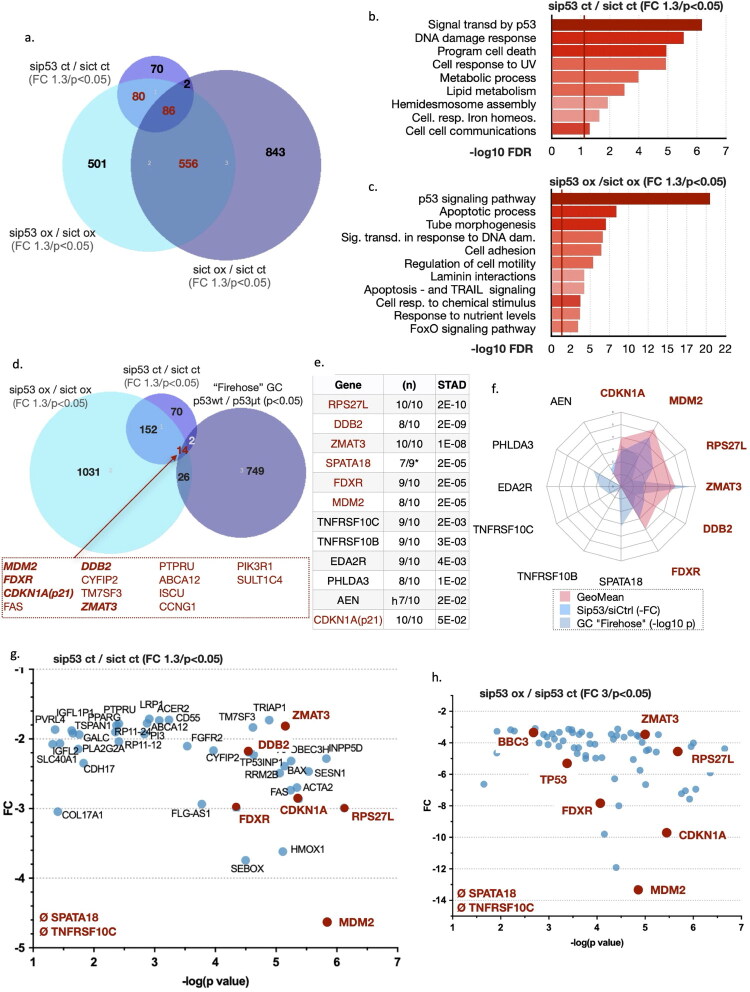
Identification and validation of p53 target genes in gastric adenocarcinoma cells. a) Venn diagram representing the overlap of differentially expressed genes (DEGs) identified in RNA-seq analysis across three conditions. Each circle corresponds to a comparative analysis between two conditions as follows: light purple circle indicates DEGs of AGS cells transfected with sip53 and non-treated (sip53 ct) versus cells transfected with the siCtrl and non-treated (sict ct) (fold change (FC) ≥ 1.3; *p* < 0.05); blue circle indicates DEGs of AGS cells transfected with sip53 and treated with oxaliplatin (sip53 ox) versus cells transfected with siCtrl and treated with oxaliplatin (sict ox) (FC ≥ 1.3; *p* < 0.05); dark purple circle indicates DEGs of AGS cells transfected with siCtrl and treated with oxaliplatin (sict ox) versus cells transfected with siCtrl and non-treated (sict ct) (FC ≥ 1.3; *p* < 0.05). **b, c**) Molecular mechanisms impacted by the genes identified in the comparison sip53 ct versus siCtrl ct (b) and the comparison sip53 ox versus siCtrl ox (c). **d**) Venn diagram as described in a) but with the overlap with the genes deregulated (*p* < 0.05) in gastric tumours present in the firehose collection of the TCGA when tumours with wild-type p53 are compared to tumours with mutated p53. **e**) Table showing the genes identified by Parikh et al.^39^ that are deregulated (*p* values under STAD) in gastric tumours (STAD) with wild-type p53 versus mutated p53. The table also indicates the number of different types of tumours that have these genes deregulated. The genes further analysed are indicated in red. **f**) Star graphic with the genes from the Tarikh et al. study showing the geomeans with the transcriptomic data (FC and *p* values for all the comparisons in AGS cells, *p* values of Tarikh et al. and *p* values of the genes differently expressed in the firehose collection between tumours with wild type p53 and mutated p53). **g**) Graphic showing the genes identified in the comparison between the sip53 ct versus the siCtrl ct conditions using the fold change (FC) and the -log(*p* values). In red, the genes of the Parikh et al. study. **h**) Graphic showing the genes identified in the comparison between the sip53 ct versus the siCtrl ct conditions using the fold change (FC) and the -log(p values). In red, the genes of the Tarikh et al. study.

We identified 248 genes that were downregulated (sip53 versus siCtrl; fold change < −1.3; *p* values < 0.05) upon silencing of p53 in the absence of treatment, and 1223 genes after treatment with oxaliplatin for 24 h (sip53 oxaliplatin versus siCtrl oxaliplatin; fold change < −1.3; *p* values < 0.05) in AGS cells (Figure 1a). Amongst them, 166 were regulated by p53 both in control conditions and after oxaliplatin treatment, 72 were only regulated in control condition, and 1057 were only regulated by p53 after oxaliplatin treatment. Oxaliplatin treatment alone induced 1399 genes (siCtrl oxaliplatin versus siCtrl; fold change > 1.3; *p* values < 0.05), of which 556 are regulated by p53. Pathway analysis of the genes regulated by p53 in untreated cells identified the expected molecular mechanisms, namely signalling transduction by p53, DNA-damage response, programmed cell death, and cell response to UV (Figure 1b). In addition, a number of uncommon pathways emerged, notably lipid metabolism, cellular response to iron metabolism, and cell-cell communication, including hemidesmosome assembly. Similarly, upon oxaliplatin treatment, the expected p53-regulated mechanisms were identified, such as p53 signalling pathway, apoptotic processes, signalling in response to DNA damage, apoptosis dependent on TRAIL signalling, and cell response to chemical stimulus (Figure 1c). Interestingly, other more unexpected pathways were also uncovered, such as tube morphogenesis, cell adhesion, laminin interaction, and response to nutrient levels including FOXO signalling.

To start the selection process of a list of genes that monitor p53 activity, we analysed the transcriptomic data of the so-called “firehose” collection of GC available within the TCGA database. We classified these tumours into two groups based on p53 status, WT or mutated. We identified 791 genes downregulated (*p* < 0.01) in p53 mutated tumours. Cross comparison of the p53-regulated genes in AGS with the p53-regulated genes in gastric tumours identified 14 common genes (*MDM2, FDXR, CDKN1A(p21), FAS, DDB2, CYFIP2, TM7SF3, ZMAT3, PTPRU, ABCA12, ISCU, CCNG1, PIK3R1, SULT1C4*) (Figure 1d). We further continued the selection process by using the data of Parhik et al.^39^ who identified genes differentially regulated according to p53 status in ten types of tumours, including GC (Figure 1e). Several of these genes (*MDM2, FDXR, CDKN1, DDB2, ZMAT3*) differentially regulated in GC were also present in our list of 14 genes (Figure 1d). We then re-analysed the published RNA-seq data from the LMSU cell line in which mutant p53 had been silenced^38^ and compared them to our data (Fig. S1b). None of the genes present in the Parikh et al. study or in our 14 genes list were present in the list of genes of the LMSU cells in which mutant p53 had been silenced, suggesting that there was no significant remaining wild-type p53 activity in LMSU cells. We then combined the data to calculate a geometric mean (GeoMean) = *i* = 1∏*nxi*)*n*1.

We converted all negative values (ex.-Log10 p) into positive values and plotted the data on a radial graph in addition to the -FC of the comparison between sip53 and siCtrl in AGS cells and the comparison between p53 mutated and p53 wild type GC of the TCGA firehose collection (Figure 1f). This allowed us to select genes with a geometric mean above 2, i.e., *CDKN1A*, *MDM2, RPS27L*, *ZMAT3*, *DDB2*, *FDXR*.

Interestingly, these genes displayed a variety of fold changes and *p* values in the RNA-seq analysis we performed, allowing us to have a diversity of responses for testing p53 activity (Figure 1f, g). We decided to complete this list of genes with *PUMA* (*BBC3*), a well-studied p53 target gene that was only expressed in the oxaliplatin-treated AGS cells and showed moderate FC and *p* values (Figure 1g, h, Fig. S1c).

**Figure 2.**
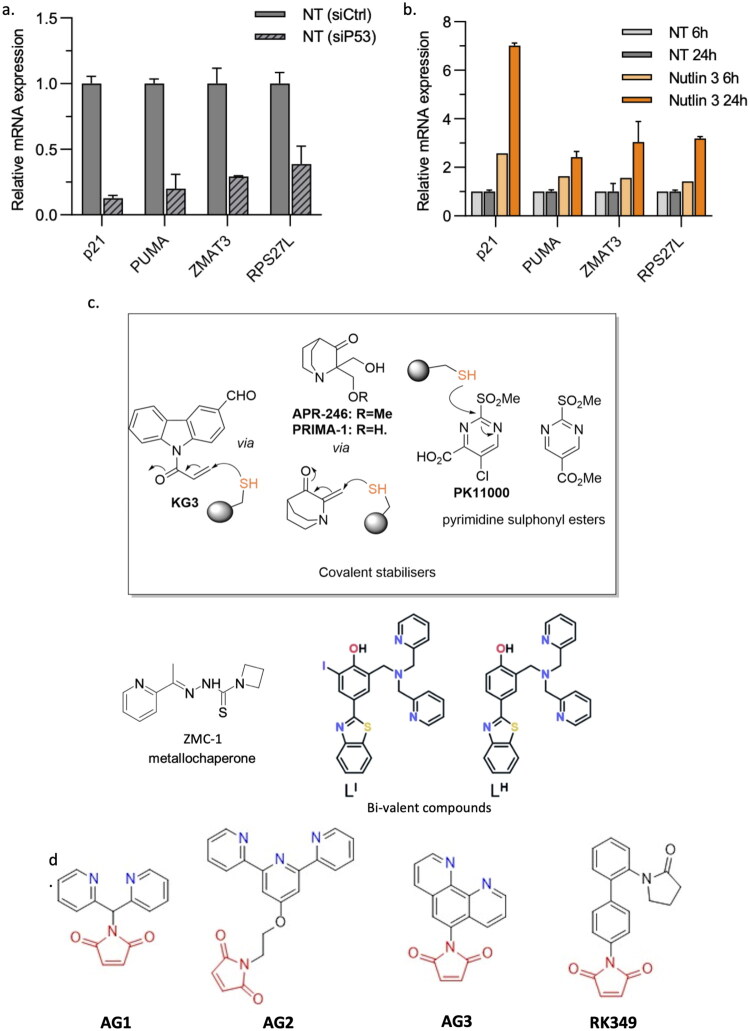
Reactivators of p53 mutants**. a**) Relative mRNA expression of selected genes after transfection of AGS cells by control (siCtrl) or p53-specific silencing RNA (siP53) for 48 h. Values obtained with siCtrl were set at 1 for each gene. **b**) Relative mRNA expression of selected genes after treatment of AGS cells with Nutlin-3 (0.4 µM) for 6 h or 24 h. Values obtained with non-treated cells at both timepoints were set at 1 for each gene. **c**) Chemical structures of covalent p53 Y220C stabilisers and a metallochaperone. **d**) Initially screened molecules for Y220C p53 reactivation.

Next, we validated in an independent experiment in AGS cells the response of the selected genes to the silencing of p53 and the activation of p53 using Nutlin-3, an antagonist of the p53-MDM2 interaction (23)(24). Nutlin-3 reduced AGS cell survival (Fig. S2a, b) and increased p53 protein levels (Fig. S2c, d). As expected, the expression of the selected genes was strongly diminished upon the silencing of p53 and activated by Nutlin-3 treatment (Figure 2a, b and Fig. S2e). The induction of the genes by Nutlin-3 was strongly dependent on p53 as the expression of p53 target genes strongly diminished upon silencing of p53 (Fig. S2e).

Based on the combined transcriptomic and *in silico* analyses and the activation by Nutlin-3, we chose to focus on *CDKN1A* (p21), *BBC3* (Puma), *ZMAT3* and *RPS27L* as indicators of the ability of a chemical compound to induce p53 activity in GC cells, and we set out to use them as a readout of p53 activity upon treatment of Y220C-mutant cells with bivalent small molecules combining a thiol-reactive warhead with a metallochaperone moiety.

### Cytotoxicity of the rationally designed compounds towards Y220C p53 and their reactivity in GC and healthy cells

Covalent p53-Y220C stabilisers (Figure 2c) have recently been described, which take advantage of the reactivity of cysteine residues and offer the potential of longer action^32^^,^^40^^,^^41^. Other studies have also identified zinc chelators as promising agents, potentially acting as metallochaperones to facilitate zinc transport into cells harbouring zinc-binding deficient p53 mutants^30^^,^^42^. More recently, we have conceived bifunctional ligands L^I^ and L^H^ to reduce the misfolding of p53 through zinc binding with more specificity^15^. Given the promise of the covalent approach, we investigated a small library of bifunctional modifiers capable of not only covalently binding to the Cys220 residue or other solvent-exposed cysteines in the DBD via a Michael acceptor but also featuring metal-chelating properties (Figure 2d).

**Figure 3.**
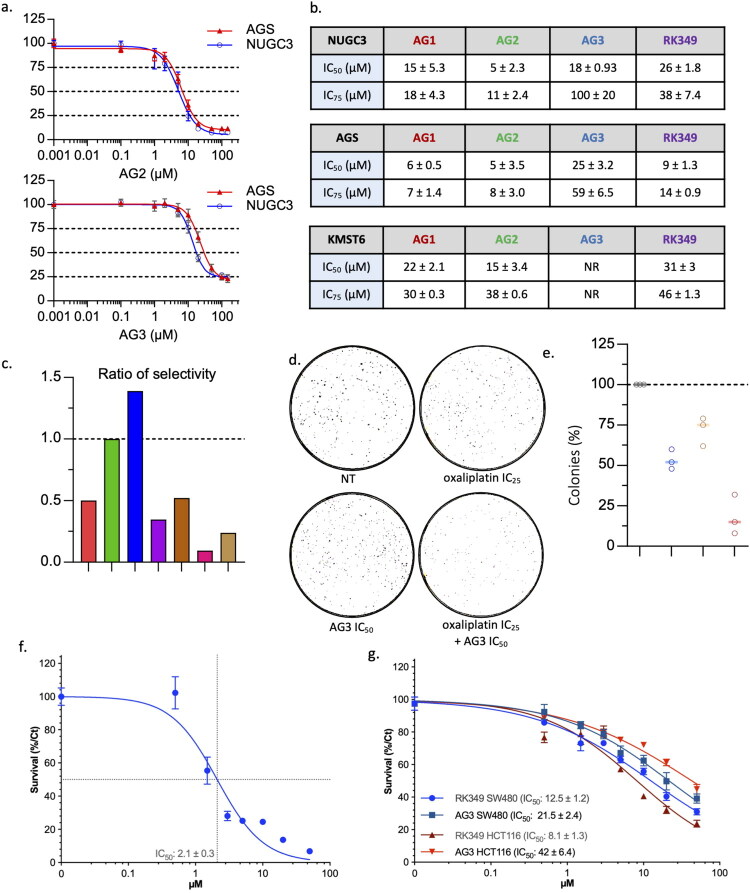
Evaluation of the cytotoxicity of compounds AG1, AG2, AG3, and RK349**. a**) MTT assays for AGS and NUGC3 cells treated with increasing concentrations of AG2 (top) and AG3 (bottom) for 48 h (*n* = 3). **b**) Mean (-/+ SD) IC_50_ and IC_75_ values of the different compounds in the indicated cell line treated for 48 h (*n* = 3). NR: not reached. **c**) Graphic representation of the selectivity of each compound using the ratio of IC_50_ for AGS (p53 WT) and NUGC3 (p53 Y220C) cells indicated in **b** and Fig S3b. **d**) Representative wells of a clonogenic assay performed with NUGC3 cells treated or not (NT) for 48 h with oxaliplatin (IC_25_, 12 µM) and/or AG3 (IC_50_, 18 µM) (*n* = 3). **e**) Quantification of clonogenic assays performed as described in **d**, with the number of colonies obtained for non-treated cells set at 100%. (*n* = 3). **f**) MTT assays for BxPC3 cells treated with increasing concentrations of AG3 for 48 h. **g**) MTT assays for HCT116 and SW480 cells treated with increasing concentrations of RK349 and AG3 for 48 h.

The cytotoxicity of AG1, AG2, AG3 and RK349 was initially tested in two widely used GC cell lines, expressing either wild-type (AGS) or mutated Y220C (NUGC3) p53 protein. Compounds designed to target mutant p53 such as ZMC1, Phikan083 and APR-246 were also tested for comparison^43–45^. Standard MTT tests were performed to define inhibitory concentrations required for an effect over 50% (IC_50_) or 75% (IC_75_) of the cells after 48 h (Figure 3a-b, Fig. S3a-b). The amount of GC cells was reduced by all the compounds tested, with AG2 exhibiting the lowest IC_50_ value in both cell lines (5 µM) and other compounds showing similar or higher IC_50_ values, depending on the cell line. Strikingly, most compounds were more efficient in AGS cells. For each compound, we quantified the variability of IC_50_ by calculating the ratio between the one in AGS and the one in NUGC3 cells (Figure 3c).

Only AG3 exhibited a ratio higher than 1 (i.e., IC_50_ lower in NUGC3 than in AGS), which may be due to a preferential effect towards cells with mutated p53. The other compounds (AG1-2 and RK349), including the ones reported to target mutated forms of p53 (ZMC1, APR-246, Phikan083), had a ratio equal or lower to 1, suggesting a lack of specificity for the Y220C mutation in GC cells. For the Y220C-binder PhiKan083 this was expected at the concentration tested, given its relatively weak Y220C-binding affinity of about 150 µM and only moderate stabilisation *in vitro*^43^ as well as the general toxicity of the carbazole-based scaffold at higher concentrations in cells^46^.

The cytotoxic effects of AG3 in NUGC3 cells were confirmed using a clonogenic assay (Figure 3d). The combination of AG3 with a low concentration of oxaliplatin (IC_25_), known to upregulate p53, was observed to increase the cytotoxicity of both molecules against NUGC3, suggesting that AG3 may boost chemotherapy for GC patients (Figure 3d**-**e, S1d).

We then confirmed the cytotoxic activity of AG3 on another GC cell line expressing the mutant Y220C, BxPC3 (Figure 3f), and on the colon cancer cell line SW480 expressing the DNA-contact mutant R273H (Figure 3g). Interestingly, AG3 showed some selectivity towards the cells expressing mutated p53 compared to the colon cancer cell line HCT116 expressing wild-type p53. This was not the case for RK349.

To characterise a novel anticancer compound, its intrinsic toxicity is investigated by treating non-tumoral cells with the compound alone. To do so, we chose the human immortalised KMST-6 fibroblasts. AG3 did not show any significant toxicity towards the latter, at the tested concentrations. In contrast, AG1, AG2 and RK349 exhibited up to three times higher IC_50_ values than on GC cells (Figure 3b, Fig S3c). Based on this first set of results and the relatively more complex synthetic procedure for RK349, we decided to focus our study on AG1, AG2, and AG3.

**Figure 4.**
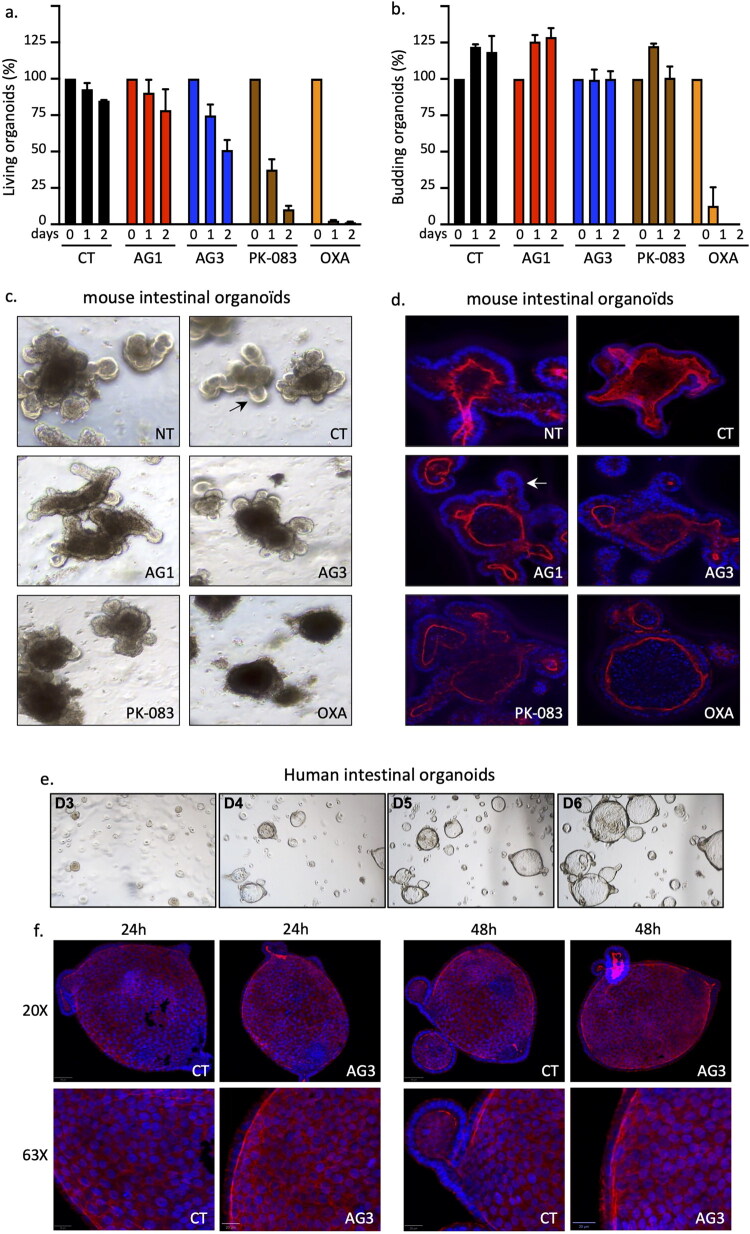
Evaluation of the cytotoxicity of the new compounds on intestinal organoids. **a**) Quantification of organoids alive after 24 h and 48 h of treatment with the vehicle (CT) or the indicated compound at the IC_50_. The initial number of organoids was set to 100% for each condition. **b**) As in panel **a** for budding organoids. **c**) Representative images taken with a bright-field microscope of untreated organoids (NT) or treated for 48 h with the indicated compound. Arrow: bud. Magnification: 5x. **d)** As in panel **c** but apical membranes (red) and nuclei (blue) of intestinal cells were stained with FITC-phalloidin (red) and DAPI (blue) respectively and imaged using an ApoTome fluorescence microscope for optical sectioning. Magnification: 20x. **e**) Representative images taken with a bright-field microscope of untreated human-derived organoids for 6 d following splitting. **f**) As in panel (**d)**, apical membranes (red) and nuclei (blue) of intestinal cells were stained with FITC-phalloidin (red) and DAPI (blue) respectively and imaged using an ApoTome fluorescence microscope for optical sectioning. Human organoids were un-treated or treated with AG3 for 24 h and 48 h at IC_50_.

To further confirm that the new compounds have fewer adverse side effects on healthy tissues than chemotherapy, we used intestinal organoids (IOs) as the gut is one of the most impacted organs by chemotherapy side effects^47^. The use of IOs offers the advantage of providing non-mutated cells in an organised tissue-like model with stem cells and various differentiated cells. In addition, it limits the use and harm to animals compared to the establishment of a maximal tolerated dose. We first performed a survival assay with mouse IOs after a treatment with AG1, AG3, oxaliplatin, and PhiKan083 at their IC_50_. In striking contrast to oxaliplatin, and to a lesser extent PhiKan083, the new compounds had only a limited effect on the survival and structure of the IOs. Indeed, after 48 h of treatment, more than 90% of IOs were dead with oxaliplatin and PhiKan083, whereas 50 to 80% were still alive (e.g., similar shape, volume compared to control) with AG3 and AG1 respectively (Figure 4a). In addition, surviving IOs were budding, meaning that new proliferative cells were generated from stem cells despite the treatment (Figure 4b**-**c, Fig. S4). Microscopic observation of the IOs’ structure confirmed the reduced impact compared to oxaliplatin (Figure 4d, Fig. S5). Similarly, we confirmed the absence of toxicity of AG3 in healthy tissues using human intestinal organoids grown in culture for 4 d before treatment (Figure 4e). At day 4, organoids were treated with AG3 at its IC_50_ for 24 or 48 h, then stained. No difference in terms of growth or cell death was observed either at 24 or 48 h (Figure 4d). Altogether, these data suggest that the new compounds have limited side effects in non-tumoral tissues.

### Ability of the new compounds to induce off-target effects through ROS

**Figure 5.**
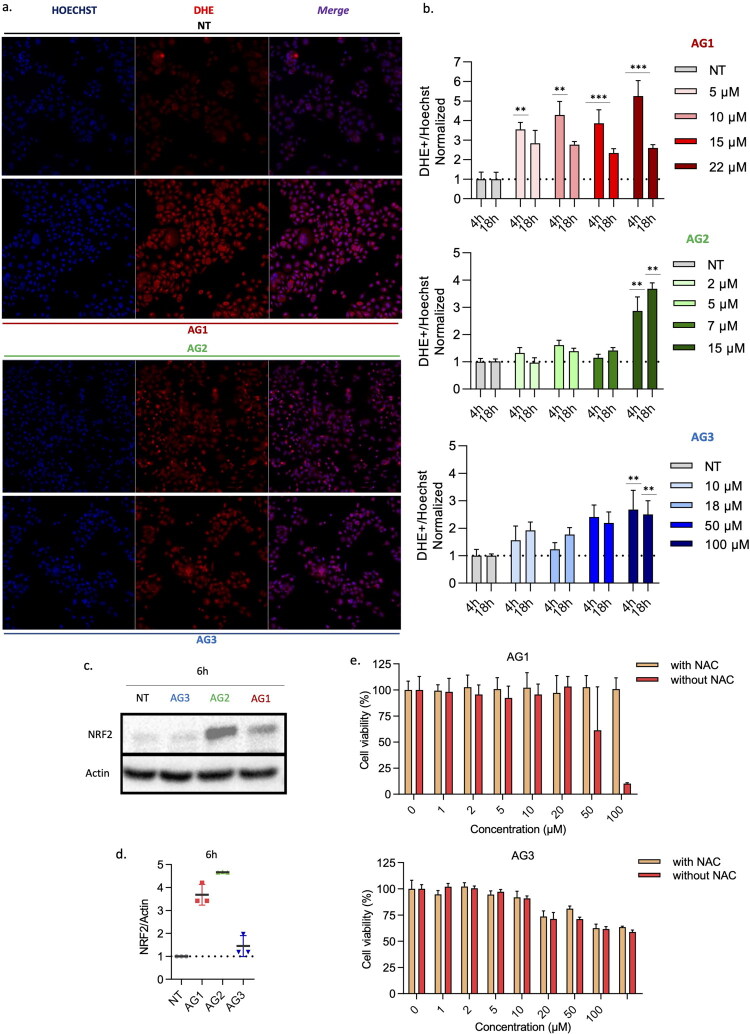
Effect of the new compounds on ROS production in p53 Y220C-mutated GC cells**. a**) Representative images taken with an ApoTome fluorescence microscope of NUGC3 cells treated for 4 h at the IC_50_ of the indicated compound. Cells were stained with dihydroethidium (DHE) to detect ROS (red) and Hoechst dye to detect nuclei (blue). **b**) Graphic representation of the ratio between DHE-positive cells and total cells after treatment with AG1, AG2, or AG3 for 4 h and 18 h at the indicated concentrations (*n* = 3). For both timepoints, the ratio for non-treated cells was set at 1. **c)** NRF2 expression detected by western blot in NUGC3 cells untreated (NT) or treated for 6 h with the indicated compound at IC_50_. Actin expression was used to control equivalent protein levels in each lane. **d)** Quantification of relative NRF2 expression levels in three independent experiments performed as in **c**, with untreated cells considered as 1. **e)** Cell viability assay for NUGC3 cells after pre-treatment (orange) or without treatment (red) with N-acetylcysteine (NAC, 1 mM) for 1 h, followed by treatment with increasing doses of the indicated compound for 48 h (*n* = 3). Values for untreated cells with or without NAC were considered as 100% viability.

Given the lack of specificity observed for most compounds towards the NUGC3 cell line expressing the p53 Y220C mutant and the known off-target effect of alkylating agents (i.e., APR-246) to cause reactive oxygen species (ROS) production, we examined ROS levels in GC cells. We used a dihydroethidium (DHE) probe to measure ROS after 4 h and 18 h of treatment at a wide range of concentrations including IC_50_ and IC_75_ (Figure 5a-b). We observed that even low doses of AG1 led to a rapid and robust production of ROS (e.g., 4- to 5-fold after 4 h). ROS production was lower for AG3, as well as AG2 (except for the highest concentration).

In addition, we analysed the expression of the anti-oxidant protein NRF2 (i.e., nuclear factor erythroid 2-related factor 2), a marker of ROS activity^48^. Increased protein level for NRF2 induced by AG1 and AG2 showed the functional impact of ROS production following these two treatments (Figure 5c-d). For the control compounds, we observed that, as described in the literature, both ZMC1 and APR-246 led to significant ROS production after 4 h of treatment at IC_50_ (Fig. S6a). This correlated with the increase in NRF2 expression for APR-246 (Fig. S6e).

To determine the importance of ROS production for the cytotoxicity of the new compounds, we performed MTT assays in the presence of the anti-oxidant *N*-acetylcysteine (NAC)^49^ (Figure 5e, Fig. S6b). Cytotoxicity of AG1 in NUGC3 cells was almost totally inhibited by NAC, confirming its strong dependence on ROS production. In contrast, little to no impact of NAC was found for cellular viability after AG2 and AG3 treatment, indicating that these compounds do not exert their effect through ROS. For the control compounds, the cytotoxicity of ZMC1 was unexpectedly not affected by NAC^50^^,^^51^. In contrast, the cytotoxicity of APR-246 was dependent on ROS production (Fig. S6b).

### Impact of the new compounds on the expression and activity of p53

**Figure 6.**
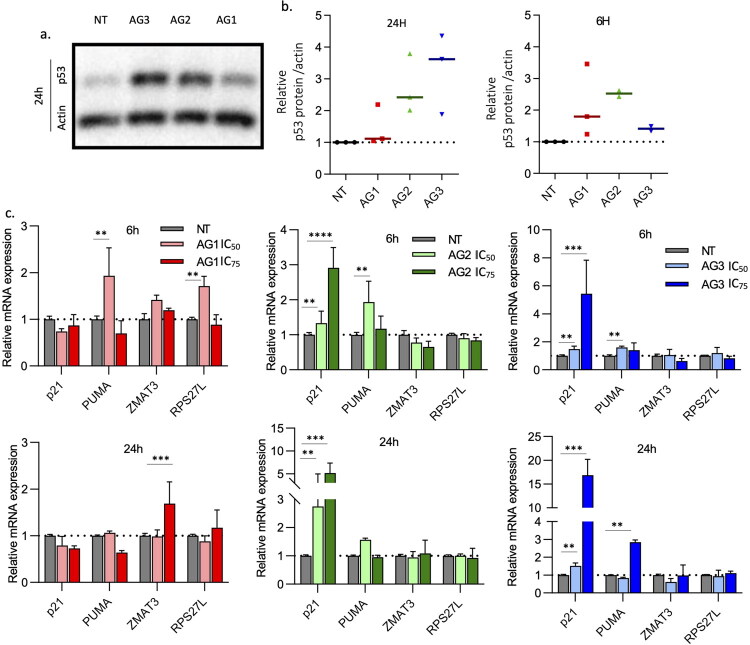
Effect of the new compounds on p53 expression and function in p53 Y220C-mutated GC cells**. a)** Western blot detection of p53 and actin proteins after treatment of NUGC3 cells with the indicated compound for 24 h. **b)** Quantification of relative p53 protein levels obtained in several experiments performed as in panel **a** and calibrated by the non-treated condition (NT) (*n* = 2–3 at 6 h and *n* = 3 at 24 h). **c)** Relative mRNA levels of *p21*, *PUMA*, *ZMAT3* and *RPS27L* after treatment with AG1, AG2 and AG3 for 6 h (top) and 24 h (bottom) at the IC_50_ and IC_75_ (*n* = 3), with non-treated cells considered as 1.

We evaluated the protein expression of p53 after 6 h and 24 h of treatment with AG1, AG2, and AG3 (Figure 6a-b). AG1 treatment resulted in a negligeable to moderate increase of p53 protein expression levels in NUGC3 cells. In contrast, p53 protein levels were consistently increased by AG2 at both time points. AG3 also induced higher levels of p53 after 24 h of treatment by an average of 3.5-fold compared to non-treated cells. Despite some heterogeneity in the results, AG compounds led to higher levels of p53 protein, suggesting higher stability of the protein and/or higher protein expression rates.

Next, we assessed the effect of the new compounds on the transcriptional activity of p53 after either 6 h or 24 h of treatment using the target genes identified by the combined *in silico/in vitro* screening method (Figure 6c). Some small changes in expression were observed upon AG1 treatment, which depended on the gene, dose and timepoint considered. In contrast, AG2 clearly increased *p21* expression at both time points as well as *PUMA* expression at 24 h. AG3 led to an even higher expression of *p21* and *PUMA* at 6 h and 24 h. We further confirmed that AG3 has the highest impact on p53 in terms of protein expression but also transcriptional activity when treating the cells for 36 h: expression of *p21* and *PUMA* were only slightly up-regulated by AG1, while a 15–30-fold increase was observed with AG3 (Fig. S7a). Hence, AG3 seems more effective than the other compounds in its ability to increase p53 transcriptional activity.

### Structural and functional determinants of the cytotoxicity of the new compounds

**Figure 7.**
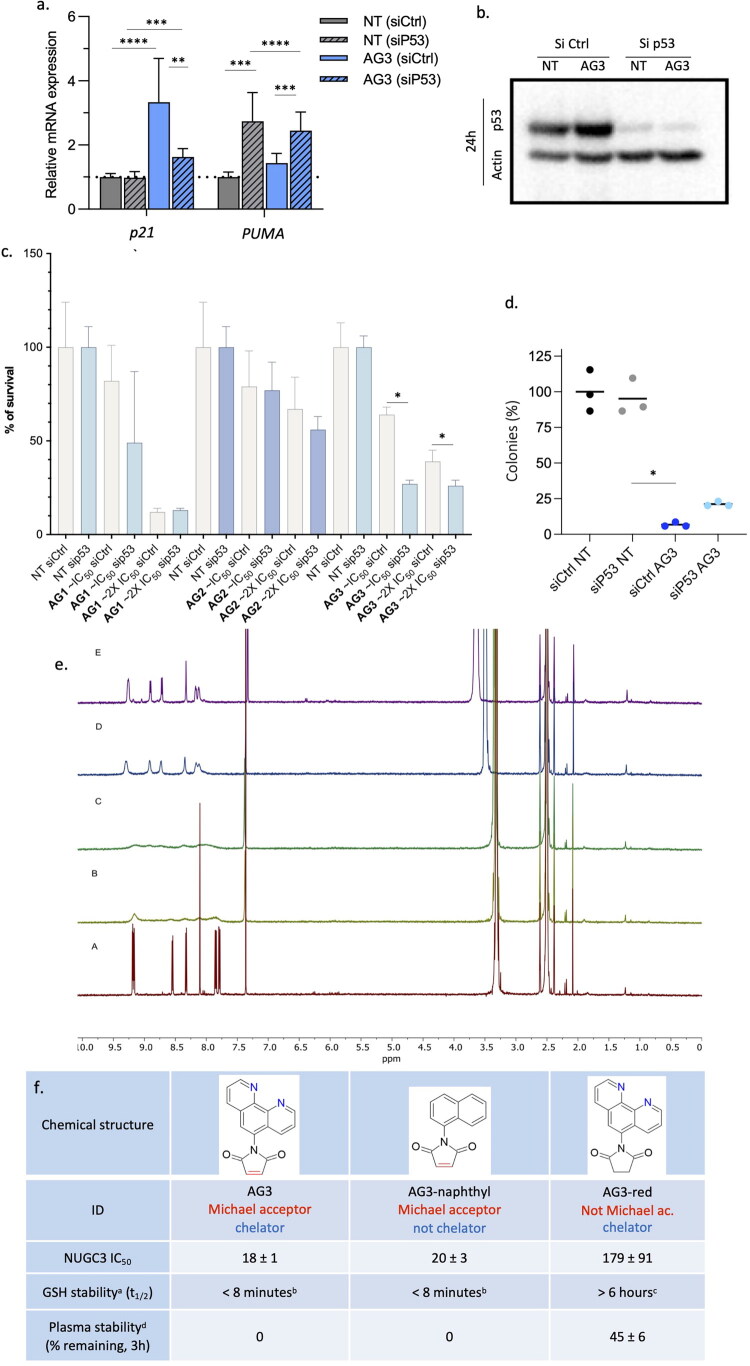
Structural and functional determinants of the cytotoxicity of the new compounds**. a)** Relative mRNA expression of *p21* and *PUMA* under the conditions described in Figure 7(e). Values obtained with untreated cells transfected with control siRNA were considered as 1 for each gene (*n* = 3). **b)** Western blot assessing p53 and actin expression in NUGC3 cells transfected for 48 h with either control (siCtrl) or p53-silencing RNA (sip53) and then treated for 24 h with AG3 (IC_50_). c) MTT assays performed with NUGC3 cells transfected with either control (siCtrl, gray) or p53-silencing RNA (sip53, blue) and treated or not (NT) for 48 h with AG1, AG2, or AG3 at the IC_50_ or 2x the IC_50_. Values obtained with untreated cells transfected with control siRNA were considered as 100% viability (*n* = 5). **d)** Clonogenic assay for NUGC3 cells transfected with control (siCtrl) or p53 (sip53) siRNA and left either untreated (NT) or treated with AG3 at the IC_50_ (*n* = 3). **e)** ^1^H NMR (600 MHz) spectra of AG3 (d_6_-DMSO) upon addition of an aqueous ZnCl_2_ solution (D_2_O) (referenced to DMSO δ 2.5 ppm). A: AG3 in d_6_-DMSO (1 mM). B: 0.5 eq. ZnCl_2_. C: 1 eq. ZnCl_2_. D: 5 eq. ZnCl_2_. E. 10 eq. ZnCl_2_. **f)** Table summarising the chemical structures of AG3 derivatives, their biological and biochemical activities. AG3-red (without the Michael acceptor property) and AG3-naphthyl (without the zinc-binding property) are represented with their respective IC_50_ in NUGC3 cells (*n* = 3). In vitro GSH and plasma stability of AG3 and its variants. Notes: ^a^ determined by ^1^H NMR in KPi buffer, pH 7.0, rt; ^b^ no alkenic protons left after 8 min; ^c^ no observable change over 6 h; ^d^ determined by LCMS versus reserpine standard, duplicate measurements.

To determine if the effect of AG3 on p53 target genes was directly dependent on p53, we silenced its expression by transfecting NUGC3 cells with an siRNA specific for p53. The increase of *p21* expression observed upon AG3 treatment for 24 h was blunted upon silencing of p53, suggesting a direct effect of AG3 on p53 (Figure 7a). It is of note that p53-silencing was also efficient upon AG3 treatment (Figure 7b). Surprisingly, the silencing of p53 increased *PUMA* expression in NUGC3 cells, whether they were treated or not with AG3, suggesting an inhibitory effect by mutated p53 that seemed slightly less important with AG3. Hence, our results are consistent with AG3 restoring the activity of the p53 Y220C mutant on a subset of target genes.

Our compounds were selected to induce cytotoxicity by restoring p53 function in tumour cells that express the p53 Y220C mutant. We assessed their functional dependence towards p53 by performing MTT assays after transfection of NUGC3 cells with control or p53 silencing RNA (Figure 7b**-**c, Fig. S7b). For AG1 and AG2, the absence of p53 had no impact on the cytotoxicity, neither at the IC_50_ nor at twice the IC_50_. For AG3, the absence of p53 caused a statistically significant reduction of cytotoxicity, indicating that AG3’s activity on the NUGC3 tumour cells depends in part on p53. This trend was also observed at additional concentrations although without statistical significance (Fig. S8). In contrast, the silencing of p53 had no impact on the cytotoxic activity of ZMC1 and APR-246, further suggesting the involvement of p53-independent mechanisms (Fig. S8). Clonogenic tests confirmed that the cytotoxic activity of AG3 depends on p53 levels as three times more colonies were observed upon p53 silencing (Figure 7d). As activation of caspases can be observed in a non-cytotoxic context^52^, we used a pan-inhibitor of caspases to assess whether AG3 induces a caspase-dependent apoptosis. The caspase (ZVAD-fmk)^53^ inhibitor reduced AG3 cytotoxicity on NUGC3 cells. Similarly, the ferroptosis inhibitor (Ferrostatin-1)^54^ reduced AG3 cytotoxicity. These results suggest that AG3 may induce apoptosis and ferroptosis (Fig. S7b).

As AG3 induced a p53-dependent cytotoxicity, we wanted to identify if either zinc chelation and/or its function as a Michael acceptor could be driving its activity. The Michael acceptor ability of AG2 and AG3 has previously been verified by mass spectrometry studies at 100 µM protein concentration^55^, whilst potentiometric and NMR studies for selected examples at millimolar concentrations of ligand and zinc indicate zinc binding in aqueous-based solutions (Figure 7e, Fig. S9a). Of note, slow ring opening of the maleimide by water was also observed both in the presence and absence of ZnCl_2_, reaching ∼ 20% after 4 h and ∼50% after 36 h (Fig. S9). Full maleimide hydrolysis to give a single product was eventually observed after multiple days (Fig. S9b). The NMR studies confirm the ability of AG3 to bind Zn, but at much higher concentrations than the application of these compounds *in vivo*, expected to be µM levels (i.e., up to 3 orders of magnitude less concentrated solutions). NMR is unsuitable for monitoring compound behaviour or complex formation at lower concentrations, and thus, it is unclear how relevant these observations are to the observed activity. Therefore, an alternative approach to understanding the role of the structural feature on the cytotoxicity of AG3 was taken.

We investigated a reduced form of AG3 (i.e., AG3-red), devoid of the Michael acceptor properties, which was synthesised alongside a naphthalene form (i.e., AG3-naphthyl) lacking the zinc-binding ability, which was commercially available (Figure 7f). MTT assays revealed that the cytotoxicity of AG3 in NUGC3 cells required its Michael acceptor property (IC_50_ AG3-red: 179 µM) but not its zinc-binding capacity (IC_50_ AG3-naphthyl: 20 µM).

### Covalent modification of the p53-Y220C DBD by AG3 and its effect on thermostability

Based on the p53-dependent cellular activity of AG3, we performed mass spectrometry and thermal shift assays by differential scanning fluorimetry (DSF) with recombinant p53 DBD to monitor the effects of AG3 on p53 alkylation and thermal stability. The Y220C mutation was introduced into a stabilised quadruple mutant variant (QM; M133L/V203A/N239Y/N268D) that we routinely used in the past for biophysical and structural studies on p53 mutants^27^^,^^56^^,^^57^. The Y220C DBD contains 11 Cys residues: three of them (C176, C238, and C242) are involved in zinc binding. Incubation of the Y220C DBD with AG3 at room temperature for 16 h (1:25 ratio, 500 µM AG3) resulted in different species of hyperalkylated Y220C mutant but also showed a significant population of nonalkylated protein (Fig. S10c). In addition to the expected mass increase of 275 Da (or multiples thereof), we also observed a mass increase of 293 Da (especially at higher temperature and longer incubation time), suggesting hydrolysis and ring opening (Fig. S10a). Control experiments with AG3-red showed no alkylation, as expected. Incubation with AG3-naphthyl under the same conditions resulted in complete alkylation of all 11 Cys in the Y220C DBD, indicating complete unfolding of the protein, which may be facilitated by the higher lipophilicity or reactivity of AG3-naphthyl (Fig. S10d). Consistent with this observation, no folding transition could be observed in DSF thermal shift assays after incubation with 500 µM AG-naphthyl (Fig. S11b). Treating WT and Y220C DBD with 10 µM AG3 had, in both cases, no effect on protein stability in DSF thermal shift assays after incubation for 1 or 19 h at RT. The unfolding curves and the derived melting temperatures (*T*_m_ values) of treated and untreated samples were virtually identical (Fig. S11a). With 50 µM AG3 a slight destabilisation was observed after 19 h incubation. At a compound concentration of 500 µM, high initial fluorescence combined with no clearly observable folding transition indicated that most of the protein had completely unfolded during incubation. A comparison of the thermal shift assays after incubation with 500 µM AG3, AG3-red, and the control without compound (Fig. S11b) suggests that unfolding of the protein at high AG3 concentrations was induced by alkylation but that the zinc chelating-moiety also contributed to destabilising the mutant. The WT protein was more stable under the same conditions.

We also investigated the intrinsic thiol reactivity of AG3, and its stability in human plasma, using previously reported protocols^58^. AG3 and AG3-naphthyl were completely depleted in under 8 min upon incubation with excess GSH (pseudo first order conditions), as monitored by ^1^H NMR spectroscopy (Figure 7f). The disappearance of the alkenic protons of the maleimide motif was particularly diagnostic, in combination with new signals consistent with Michael adduct formation visible in the aliphatic region. In contrast, AG3-red remained completely stable over 6 h under the same conditions, consistent with its lower reactivity. The trends were similar when the same three compounds were incubated with human plasma. We could not detect AG3 and AG3-naphthyl by LCMS analysis after 3 h incubation in plasma, while approximately half of AG3-red was depleted in the same conditions. Altogether, these data confirmed the high thiol reactivity of maleimide derivatives AG3 and AG3-naphthyl, and suggest that this reactivity is also the reason for their much-diminished stability in plasma.

## Discussion

The discovery of new anticancer agents that can selectively target GC cells, overcome drug resistance, and work synergistically with existing treatments may revolutionise the management of this disease. Advances in genomics, proteomics, and bioinformatics are paving the way for the identification of novel drug targets and the development of precision medicine. In this regard, mutated p53 represents an ideal target as it is present only in cancerous cells and is a root cause of cancer. However, such a p53-based strategy requires a protocol that can efficiently and precisely monitor p53 activity, despite its immense complexity, which is both context-dependent (cell type) and stimulus-dependent (e.g., after DNA damage or nutrient depletion).

### A subset of p53 target genes to evaluate p53 activity in gastric cancer

To define a subset of p53 target genes that may best represent p53 activity in GC cells, we used an *in silico* strategy combining multiple transcriptomic data sets from cell lines as well as from patients’ tumour samples expressing wild-type or mutated p53. Our analysis highlights the difficulty of finding a robust subset of p53 target genes that may represent the activity of p53 in GC cells. For instance, the study of Parikh et al. on tumours with wild-type p53 and mutated p53 identified 12 genes that were differently expressed recurrently in 10 different types of cancers including GC. However, our analysis of the “firehose” collection of gastric tumours of the TCGA confirmed only 8 of them (*p* values < 0.05). In addition, two of them, *SPATA18* and *TNFRSF10C_,_* were neither detectable in AGS (wild-type p53) nor in LMSU (mutated p53) GC cells. Hence, to identify p53 target genes for evaluating wild-type p53 activity in GC cells, we used RNA-seq on AGS cells treated with oxaliplatin, which induces DNA damage and p53 ^12^, and used siRNA to silence p53 expression. This allowed us to identify 642 genes that are both regulated by p53 and induced by oxaliplatin, representing 43% of the genes induced by oxaliplatin. As expected, the genes regulated by p53 in AGS GC cells are controlling cell death (e.g., *BAX*, *PUMA*, *FAS, BIK*) and are involved in the p53 pathway (e.g., *MDM2*, *ZMAT3, RPS27L*). Lesser-known mechanisms regulated by p53 were also unveiled by this analysis, such as lipid metabolism (*LRP1*, *RETSAT*), iron metabolism (*FDXR*) and cell adhesion (e.g., *CDH17*, *LAMA3*). After comparing the published data set and our analysis of the TCGA, we selected 6 genes (*CDKN1*, *RPS27L*, *ZMAT3*, *FDXR*, *MDM2* and *BBC3*) to be further tested. Based on their expression levels in RT-qPCR experiments (i.e., representative of different levels of expression) and induction by the p53 activator Nutlin-3, we chose for routine screening the genes *CDKN1A*, *RPS27L*, *ZMAT3,* and *BBC3*, representative of the p53 functions in cell-cycle arrest, auto-regulation and apoptosis. Obviously, the final choice was somewhat subjective as it could have also included other genes (e.g., *FDRX* and *MDM2)*, but screening the activity of novel compounds at various time points and concentrations for 4 genes is already more than the norm. In the end, we do believe that *CDKN1A*, *RPS27L*, *ZMAT3* and *BBC3* represent a pertinent subset of p53 target genes to assess p53 activity in GC cells.

### Reactivation of Y220C p53 in NUGC3 gastric cancer cells? AG3 as a hit

To investigate the possible rescue of the p53 mutant Y220C in GC cells, we used as “positive” controls several compounds validated in the literature as p53-rescue treatments *in vitro* (PhiKan083) or in cells (APR-246, ZMC-1), and performed cytotoxicity and ROS production assays with them. Our results indicate that these molecules do not have a significant selectivity towards the cells expressing the Y220C p53 mutant. For the confirmed Y220C-binder PhiKan083, this is easily explained by its relatively high binding constant of 150 µM. In previous work, we showed that more potent, second-generation Y220C binders become effective in NUGC3 cells once a certain threshold affinity is reached^59^. The tested molecules showed significant off-target ROS production, and the efficacy of APR-246 was dependent on ROS, as seen previously^60^. To our knowledge, this is the first evaluation of some of these molecules (i.e., APR-246, ZMC1) in GC. The lack of selectivity may explain the recent clinical outcome of APR-246. More recently, the Y220C stabiliser Rezatapopt has been developed, which is currently in a clinical trial targeting Y220C-mutant solid tumours (NCT04585750) and was shown to inhibit the growth of NUGC3 tumour xenografts in nude mice^61^^,^^62^. Unfortunately, Rezatapopt was not available for use as a control at the time of the experiments.

In this study, we have tested four novel compounds that were potential candidates to reactivate the Y220C p53 mutant based on their chemical characteristics. Out of the four, only AG3 showed a reasonable selectivity towards GC cells expressing the Y220C mutant, which was confirmed for two different GC cell lines (NUGC3 and BxPC3). The three other compounds, AG1, AG2, and RK349, were more efficient in cells expressing wild-type p53. In addition, AG1 and AG2 induced higher levels of off-target ROS production and were more toxic towards non-transformed cells or intestine organoids that contain multiple healthy cell types. It is important to note that AG3 also displayed toxicity towards colon cancer cells expressing the DNA-contact mutant R273H, indicating that the AG3-induced effects are not mutant-specific. These effects cannot be solely attributed to modulation of p53’s conformational stability, as reactivation of R273H through simple thermal stabilisation is mechanistically impossible.

AG3 shows interesting additional features pointing to its potential value as a “hit” for reactivating p53 function in Y220C p53 GC cells. Firstly, AG3 is less toxic to organoids, and its toxicity is independent of ROS production. Secondly, AG3 is also able to increase the expression of two p53 target genes, *CDKN1A* (p21) and *BBC3* (Puma), over time (6 h, 24 h and 36 h). Importantly, the cytotoxicity of AG3 in Y220C-p53 expressing cells is, in part, dependent on the expression of p53 at given concentrations (IC_50_ and 2x IC_50_). Finally, AG3 synergizes with oxaliplatin in GC cells expressing Y220C p53.

However, although these results are encouraging, it is important to also note several points that highlight the limits of AG3, which may also be the absolute limit of this approach. For instance, AG3 does not increase the expression of the two other p53 target genes we tested, *ZMAT3* and *RPS27L*. In addition, only the expression of *CDKN1A* seems dependent on the expression of p53. These somewhat unsatisfying results highlight the complexity of the p53 signalling pathways and how p53 regulates gene expression via subtle changes in p53 conformation following post-translational modifications. One possible hypothesis is that AG3, or other putative p53-reactivating compounds, may modify the protein to an extent that enables only the binding and activation of a subset of p53 target genes (i.e., *CDKN1A* and *BBC3* in the case of AG3). This hypothesis is supported by the observation that pifithrin-α differently affects various p53 target genes following specific post-translational modifications of p53^63^. Alternatively, despite the significantly reduced thermodynamic stability and shorter half-life of unfolding of the Y220C mutant, there seems to be a small fraction of correctly folded mutant protein in cells, as seen in immunofluorescence studies with conformation-specific antibodies in HUH7 liver cancer cells^64^, that is however not sufficient to induce a full p53 response. Nevertheless, it appears to be sufficient to cause p53-dependent cytotoxicity, as demonstrated using p53 siRNA.

Additional investigations are required to fully understand how AG3 induces the p53 response, as for now the only evidence available is the increase of p53 protein levels in NUGC3 cells. This increase is transcription-independent as AG3 did not elevate p53 mRNA levels (data not shown). Hence, AG3 may somehow impact Y220C-p53 translation or stability. The latter might be mediated by the covalent modification of a fraction of the p53-Y220C DBD caused by AG3. It will be interesting to study the alkylation patterns of the protein under the cellular conditions where a cytotoxic effect is seen. We did not observe a thermostabilization upon AG3 treatment of recombinant Y220C mutant DBD protein at a concentration of 10 µM as hyperalkylation induced unfolding. It is however conceivable that partial alkylation of the most reactive surface-exposed cysteines of Y220C and other mutants under specific cellular conditions may result in moderate thermostabilization, similarly to what was observed for recombinant mutant p53 protein with sulfonylpyrimidines^41^ or APR-246^20^.

It is also important to point out that the cytotoxicity of AG3 is only partially dependent on the expression of p53 in NUGC3 cells, and that this is statistically relevant only at certain concentrations (IC_50_ and 2x IC_50_). It is possible that at lower concentrations, AG3 is not concentrated enough inside the cells to elicit a p53 response. At higher concentrations, it is likely that off-target effects of AG3 take over, common to all pharmacologically active molecules. Nevertheless, AG3 appears to be an interesting starting compound to be further optimised.

## Conclusion

In summary, this study identified AG3 as a small molecule chemical tool for targeting GC cell lines expressing the p53 mutant Y220C. The exciting biological properties of AG3 are somewhat diminished by a risk, yet unsurprising, of short half-life in plasma due to its thiols reactivity. Future studies will aim to address these possible pharmacokinetic shortcomings, focusing on properties and stability optimisation, and possibly formulation^65^. This characterisation also contributed to setting up a screening strategy by providing a list of p53 target genes suitable for assessing p53 function in GC and a system to assess the off-target and potential side effects of such molecules. This will help in tackling the major challenge of treating more efficiently GC patients by providing a small chemical compound capable of partial restoration of p53 function while preserving a positive balance with off-target effects.

## Supplementary Material

Supplemetary materials VF-cl.docx

## Data Availability

All data are available on demand; transcriptomic data will be on the servers (EMBL-EBI ArrayExpress servers). The ESI contains primer and RNA sequences, protein expression and purification details, analytical methods; mass spectrometry, DSF, GSH off-target analyses. For chemistry, NMR and synthetic details, scanned spectra as well as stability studies are detailed.

## References

[1] Lane D, Levine A. p53 Research: the past thirty years and the next thirty years. Cold Spring Harb Perspect Biol. 2010;2(12):a000893. 20463001 10.1101/cshperspect.a000893 PMC2982174

[2] Joerger AC, Stiewe T, Soussi T. TP53: the unluckiest of genes? Cell Death Differ. 2025;32(2):219–224. 39443700 10.1038/s41418-024-01391-6 PMC11803090

[3] Leroy B, Anderson M, Soussi T. TP53 mutations in human cancer: database reassessment and prospects for the next decade. Hum Mutat. 2014;35(6):672–688. 24665023 10.1002/humu.22552

[4] Baugh EH, Ke H, Levine AJ, Bonneau RA, Chan CS. Why are there hotspot mutations in the TP53 gene in human cancers? Cell Death Differ. 2018;25(1):154–160. 29099487 10.1038/cdd.2017.180 PMC5729536

[5] de Andrade KC, Lee EE, Tookmanian EM, Kesserwan CA, Manfredi JJ, Hatton JN, Loukissas JK, Zavadil J, Zhou L, Olivier M, et al. The TP53 database: transition from the international agency for research on Cancer to the US national cancer institute. Cell Death Differ. 2022;29(5):1071–1073. 35352025 10.1038/s41418-022-00976-3 PMC9090805

[6] Joerger AC, Fersht AR. Structure-function-rescue: the diverse nature of common p53 cancer mutants. Oncogene. 2007;26(15):2226–2242. 17401432 10.1038/sj.onc.1210291

[7] Xu J, Reumers J, Couceiro JR, De Smet F, Gallardo R, Rudyak S, Cornelis A, Rozenski J, Zwolinska A, Marine JC, et al. Gain of function of mutant p53 by coaggregation with multiple tumor suppressors. Nat Chem Biol. 2011;7(5):285–295. 21445056 10.1038/nchembio.546

[8] Di Como CJ, Gaiddon C, Prives C. p73 function is inhibited by tumor-derived p53 mutants in mammalian cells. Mol Cell Biol. 1999;19(2):1438–1449. 9891077 10.1128/mcb.19.2.1438 PMC116072

[9] Oren M, Rotter V. Mutant p53 gain-of-function in cancer. Cold Spring Harb Perspect Biol. 2010;2(2):a001107. 20182618 10.1101/cshperspect.a001107 PMC2828285

[10] Ferraiuolo M, Verduci L, Blandino G, Strano S. Mutant p53 protein and the hippo transducers YAP and TAZ: a critical oncogenic node in human cancers. Int J Mol Sci. 2017;18(5):961. 28467351 10.3390/ijms18050961 PMC5454874

[11] Blanchet A, Bourgmayer A, Kurtz JE, Mellitzer G, Gaiddon C. Isoforms of the p53 family and gastric cancer: a menage a trois for an unfinished affair. Cancers (Basel). 2021;13(4):916. 33671606 10.3390/cancers13040916 PMC7926742

[12] Spaety ME, Gries A, Badie A, Venkatasamy A, Romain B, Orvain C, Yanagihara K, Okamoto K, Jung AC, Mellitzer G, et al. HDAC4 levels control sensibility toward cisplatin in gastric cancer via the p53-p73/BIK pathway. Cancers (Basel). 2019;11(11):1747. 31703394 10.3390/cancers11111747 PMC6896094

[13] Gaiddon C, Gross I, Meng X, Sidhoum M, Mellitzer G, Romain B, Delhorme JB, Venkatasamy A, Jung AC, Pfeffer M. Bypassing the resistance mechanisms of the tumor ecosystem by targeting the endoplasmic reticulum stress pathway using ruthenium- and osmium-based organometallic compounds: an exciting long-term collaboration with dr. michel pfeffer. Molecules. 2021;26(17):5386. 34500819 10.3390/molecules26175386 PMC8434532

[14] Riegel G, Orvain C, Recberlik S, Spaety ME, Poschet G, Venkatasamy A, Yamamoto M, Nomura S, Tsukamoto T, Masson M, et al. The unfolded protein response-glutathione metabolism axis: A novel target of a cycloruthenated complexes bypassing tumor resistance mechanisms. Cancer Lett. 2024;585:216671. 38290658 10.1016/j.canlet.2024.216671

[15] Miller JJ, Blanchet A, Orvain C, Nouchikian L, Reviriot Y, Clarke RM, Martelino D, Wilson D, Gaiddon C, Storr T. Bifunctional ligand design for modulating mutant p53 aggregation in cancer. Chem Sci. 2019;10(46):10802–10814. 32055386 10.1039/c9sc04151f PMC7006507

[16] Joerger AC, Fersht AR. The p53 pathway: origins, inactivation in cancer, and emerging therapeutic approaches. Annu Rev Biochem. 2016;85(1):375–404. 27145840 10.1146/annurev-biochem-060815-014710

[17] Miller JJ, Gaiddon C, Storr T. A balancing act: using small molecules for therapeutic intervention of the p53 pathway in cancer. Chem Soc Rev. 2020;49(19):6995–7014. 32869798 10.1039/d0cs00163e

[18] Benosman S, Gross I, Clarke N, Jochemsen AG, Okamoto K, Loeffler JP, Gaiddon C. Multiple neurotoxic stresses converge on MDMX proteolysis to cause neuronal apoptosis. Cell Death Differ. 2007;14(12):2047–2057. 17823617 10.1038/sj.cdd.4402216

[19] Benosman S, Meng X, Von Grabowiecki Y, Palamiuc L, Hritcu L, Gross I, Mellitzer G, Taya Y, Loeffler JP, Gaiddon C. Complex regulation of p73 isoforms after alteration of amyloid precursor polypeptide (APP) function and DNA damage in neurons. J Biol Chem. 2011;286(50):43013–43025. 22002055 10.1074/jbc.M111.261271 PMC3234838

[20] Zhang Q, Bykov VJN, Wiman KG, Zawacka-Pankau J. APR-246 reactivates mutant p53 by targeting cysteines 124 and 277. Cell Death Dis. 2018;9(5):439. 29670092 10.1038/s41419-018-0463-7 PMC5906465

[21] Degtjarik O, Golovenko D, Diskin-Posner Y, Abrahmsén L, Rozenberg H, Shakked Z. Structural basis of reactivation of oncogenic p53 mutants by a small molecule: methylene quinuclidinone (MQ). Nat Commun. 2021;12(1):7057. 34862374 10.1038/s41467-021-27142-6 PMC8642532

[22] Maslah N, Salomao N, Drevon L, Verger E, Partouche N, Ly P, Aubin P, Naoui N, Schlageter MH, Bally C, et al. Synergistic effects of PRIMA-1(Met) (APR-246) and 5-azacitidine in TP53-mutated myelodysplastic syndromes and acute myeloid leukemia. Haematologica. 2020;105(6):1539–1551. 31488557 10.3324/haematol.2019.218453 PMC7271596

[23] Wang Z, Hu H, Heitink L, Rogers K, You Y, Tan T, Suen CLW, Garnham A, Chen H, Lieschke E, et al. The anti-cancer agent APR-246 can activate several programmed cell death processes to kill malignant cells. Cell Death Differ. 2023;30(4):1033–1046. 36739334 10.1038/s41418-023-01122-3 PMC10070280

[24] Yu X, Blanden A, Tsang AT, Zaman S, Liu Y, Gilleran J, Bencivenga AF, Kimball SD, Loh SN, Carpizo DR. Thiosemicarbazones functioning as zinc metallochaperones to reactivate mutant p53. Mol Pharmacol. 2017;91(6):567–575. 28320780 10.1124/mol.116.107409 PMC5438133

[25] Chen S, Wu JL, Liang Y, Tang YG, Song HX, Wu LL, Xing YF, Yan N, Li YT, Wang ZY, et al. Arsenic trioxide rescues structural p53 mutations through a cryptic allosteric site. Cancer Cell. 2021;39(2):225–239.e8. 33357454 10.1016/j.ccell.2020.11.013

[26] Tang Y, Song H, Wang Z, Xiao S, Xiang X, Zhan H, Wu L, Wu J, Xing Y, Tan Y, et al. Repurposing antiparasitic antimonials to noncovalently rescue temperature-sensitive p53 mutations. Cell Rep. 2022;39(2):110622. 35417717 10.1016/j.celrep.2022.110622

[27] Bauer MR, Krämer A, Settanni G, Jones RN, Ni X, Khan Tareque R, Fersht AR, Spencer J, Joerger AC. Targeting cavity-creating p53 cancer mutations with small-molecule stabilizers: the Y220X paradigm. ACS Chem Biol. 2020;15(3):657–668. 31990523 10.1021/acschembio.9b00748 PMC7307883

[28] Bouaoun L, Sonkin D, Ardin M, Hollstein M, Byrnes G, Zavadil J, Olivier M. TP53 variations in human cancers: new lessons from the IARC TP53 database and genomics data. Hum Mutat. 2016;37(9):865–876. 27328919 10.1002/humu.23035

[29] Joerger AC, Ang HC, Fersht AR. Structural basis for understanding oncogenic p53 mutations and designing rescue drugs. Proc Natl Acad Sci U S A. 2006;103(41):15056–15061. 17015838 10.1073/pnas.0607286103 PMC1635156

[30] Stephenson Clarke JR, Douglas LR, Duriez PJ, Balourdas DI, Joerger AC, Khadiullina R, Bulatov E, Baud MGJ. Discovery of nanomolar-affinity pharmacological chaperones stabilizing the oncogenic p53 mutant Y220C. ACS Pharmacol Transl Sci. 2022;5(11):1169–1180. 36407959 10.1021/acsptsci.2c00164 PMC9667543

[31] Bauer MR, Jones RN, Tareque RK, Springett B, Dingler FA, Verduci L, Patel KJ, Fersht AR, Joerger AC, Spencer J. A structure-guided molecular chaperone approach for restoring the transcriptional activity of the p53 cancer mutant Y220C. Future Med Chem. 2019;11(19):2491–2504. 31633398 10.4155/fmc-2019-0181 PMC6803818

[32] Guiley KZ, Shokat KM. A small molecule reacts with the p53 somatic mutant Y220C to rescue wild-type thermal stability. Cancer Discov. 2023;13(1):56–69. 36197521 10.1158/2159-8290.CD-22-0381 PMC9827106

[33] Khan Tareque R, Hassell-Hart S, Krojer T, Bradley A, Velupillai S, Talon R, Fairhead M, Day IJ, Bala K, Felix R, et al. Deliberately losing control of C-H activation processes in the design of small-molecule-fragment arrays targeting peroxisomal metabolism. ChemMedChem. 2020;15(24):2513–2520. 32812371 10.1002/cmdc.202000543

[34] Madern N, Queyriaux N, Chevalley A, Ghasemi M, Nicolotti O, Ciofini I, Mangiatordi GF, Salmain M. Piano-stool d6-rhodium(III) complexes of chelating pyridine-based ligands and their papain bioconjugates for the catalysis of transfer hydrogenation of aryl ketones in aqueous medium. J Mol Catal B: Enzym. 2015;122:314–322.

[35] Zhang C, Srivastava P, Ellis-Guardiola K, Lewis JC. Manganese terpyridine artificial metalloenzymes for benzylic oxygenation and olefin epoxidation. Tetrahedron. 2014;70(27-28):4245–4249. 24904188 10.1016/j.tet.2014.03.008 PMC4041119

[36] Trammell SA, Goldston HM, Jr. Tran PT, Tender LM, Conrad DW, Benson DE, Hellinga HW. Synthesis and characterization of a ruthenium(II)-based redox conjugate for reagentless biosensing. Bioconjug Chem. 2001;12(4):643–647. 11459471 10.1021/bc010022q

[37] Sato T, Stange DE, Ferrante M, Vries RG, Van Es JH, Van den Brink S, Van Houdt WJ, Pronk A, Van Gorp J, Siersema PD, et al. Long-term expansion of epithelial organoids from human colon, adenoma, adenocarcinoma, and Barrett’s epithelium. Gastroenterology. 2011;141(5):1762–1772. 21889923 10.1053/j.gastro.2011.07.050

[38] Sethi N, Kikuchi O, McFarland J, Zhang Y, Chung M, Kafker N, Islam M, Lampson B, Chakraborty A, Kaelin WG, Jr., et al. Mutant p53 induces a hypoxia transcriptional program in gastric and esophageal adenocarcinoma. JCI Insight. 2019;4(15):e128439 10.1172/jci.insight.128439 PMC6693823 31391338

[39] Parikh N, Hilsenbeck S, Creighton CJ, Dayaram T, Shuck R, Shinbrot E, Xi L, Gibbs RA, Wheeler DA, Donehower LA. Effects of TP53 mutational status on gene expression patterns across 10 human cancer types. J Pathol. 2014;232(5):522–533. 24374933 10.1002/path.4321 PMC4362779

[40] Klett T, Stahlecker J, Schwer M, Jaag SJ, Masberg B, Knappe C, Lämmerhofer M, Stehle T, Boeckler FM. S(N)Ar reactive pyrazine derivatives as p53-Y220C cleft binders with diverse binding modes. Drug Des Devel Ther. 2025;19:4727–4753. 10.2147/DDDT.S513792 PMC12212008 40599607

[41] Bauer MR, Joerger AC, Fersht AR. 2-Sulfonylpyrimidines: Mild alkylating agents with anticancer activity toward p53-compromised cells. Proc Natl Acad Sci U S A. 2016;113(36):E5271–5280. 27551077 10.1073/pnas.1610421113 PMC5018792

[42] Miller JJ, Orvain C, Jozi S, Clarke RM, Smith JR, Blanchet A, Gaiddon C, Warren JJ, Storr T. Multifunctional compounds for activation of the p53-Y220C mutant in cancer. Chemistry. 2018;24(67):17734–17742. 30230059 10.1002/chem.201802677

[43] Boeckler FM, Joerger AC, Jaggi G, Rutherford TJ, Veprintsev DB, Fersht AR. Targeted rescue of a destabilized mutant of p53 by an in silico screened drug. Proc Natl Acad Sci U S A. 2008;105(30):10360–10365. 18650397 10.1073/pnas.0805326105 PMC2492497

[44] Lambert JMR, Gorzov P, Veprintsev DB, Söderqvist M, Segerbäck D, Bergman J, Fersht AR, Hainaut P, Wiman KG, Bykov VJN. PRIMA-1 reactivates mutant p53 by covalent binding to the core domain. Cancer Cell. 2009;15(5):376–388. 19411067 10.1016/j.ccr.2009.03.003

[45] Blanden AR, Yu X, Wolfe AJ, Gilleran JA, Augeri DJ, O’Dell RS, Olson EC, Kimball SD, Emge TJ, Movileanu L, et al. Synthetic metallochaperone ZMC1 rescues mutant p53 conformation by transporting zinc into cells as an ionophore. Mol Pharmacol. 2015;87(5):825–831. 25710967 10.1124/mol.114.097550 PMC4407733

[46] Luparello C, Cruciata I, Joerger AC, Ocasio CA, Jones R, Tareque RK, Bagley MC, Spencer J, Walker M, Austin C, et al. Genotoxicity and epigenotoxicity of carbazole-derived molecules on MCF-7 breast cancer cells. Int J Mol Sci. 2021;22(7):3410. 33810274 10.3390/ijms22073410 PMC8038095

[47] Sato T, Vries RG, Snippert HJ, van de Wetering M, Barker N, Stange DE, van Es JH, Abo A, Kujala P, Peters PJ, et al. Single Lgr5 stem cells build crypt-villus structures in vitro without a mesenchymal niche. Nature. 2009;459(7244):262–265. 19329995 10.1038/nature07935

[48] Kasai S, Shimizu S, Tatara Y, Mimura J, Itoh K. Regulation of Nrf2 by mitochondrial reactive oxygen species in physiology and pathology. Biomolecules. 2020;10(2):320. 32079324 10.3390/biom10020320 PMC7072240

[49] Xie C, Yi J, Lu J, Nie M, Huang M, Rong J, Zhu Z, Chen J, Zhou X, Li B, et al. N-acetylcysteine reduces ROS-mediated oxidative DNA damage and PI3K/Akt pathway activation induced by helicobacter pylori infection. Oxid Med Cell Longev. 2018;2018(1):1874985. 29854076 10.1155/2018/1874985 PMC5944265

[50] Yu X, Blanden AR, Narayanan S, Jayakumar L, Lubin D, Augeri D, Kimball SD, Loh SN, Carpizo DR. Small molecule restoration of wildtype structure and function of mutant p53 using a novel zinc-metallochaperone based mechanism. Oncotarget. 2014;5(19):8879–8892. 25294809 10.18632/oncotarget.2432 PMC4253404

[51] Zaman S, Yu X, Bencivenga AF, Blanden AR, Liu Y, Withers T, Na B, Blayney AJ, Gilleran J, Boothman DA, et al. Combinatorial therapy of zinc metallochaperones with mutant p53 reactivation and diminished copper binding. Mol Cancer Ther. 2019;18(8):1355–1365. 31196889 10.1158/1535-7163.MCT-18-1080 PMC6677634

[52] Eskandari E, Eaves CJ. Paradoxical roles of caspase-3 in regulating cell survival, proliferation, and tumorigenesis. J Cell Biol. 2022;221(6):e202201159 10.1083/jcb.202201159 PMC9106709 35551578

[53] Zhu H, Fearnhead HO, Cohen GM. An ICE-like protease is a common mediator of apoptosis induced by diverse stimuli in human monocytic THP.1 cells. FEBS Lett. 1995;374(2):303–308. 7589559 10.1016/0014-5793(95)01116-v

[54] Dixon SJ, Lemberg KM, Lamprecht MR, Skouta R, Zaitsev EM, Gleason CE, Patel DN, Bauer AJ, Cantley AM, Yang WS, et al. Ferroptosis: an iron-dependent form of nonapoptotic cell death. Cell. 2012;149(5):1060–1072. 22632970 10.1016/j.cell.2012.03.042 PMC3367386

[55] Doble MV, Jarvis AG, Ward AC, Colburn JD, Götze JP, Bühl M, Kamer PCJ. Artificial metalloenzymes as catalysts for oxidative lignin degradation. ACS Sustainable Chem Eng. 2018;6(11):15100–15107.

[56] Joerger AC, Allen MD, Fersht AR. Crystal structure of a superstable mutant of human p53 core domain. Insights into the mechanism of rescuing oncogenic mutations. J Biol Chem. 2004;279(2):1291–1296. 14534297 10.1074/jbc.M309732200

[57] Balourdas D-I, Markl AM, Krämer A, Settanni G, Joerger AC. Structural basis of p53 inactivation by cavity-creating cancer mutations and its implications for the development of mutant p53 reactivators. Cell Death Dis. 2024;15(6):408. 38862470 10.1038/s41419-024-06739-x PMC11166945

[58] Pichon MM, Drelinkiewicz D, Lozano D, Moraru R, Hayward LJ, Jones M, McCoy MA, Allstrum-Graves S, Balourdas DI, Joerger AC, et al. Structure-reactivity studies of 2-sulfonylpyrimidines allow selective protein arylation. Bioconjug Chem. 2023;34(9):1679–1687. 37657082 10.1021/acs.bioconjchem.3c00322 PMC10515483

[59] Baud MGJ, Bauer MR, Verduci L, Dingler FA, Patel KJ, Horil Roy D, Joerger AC, Fersht AR. Aminobenzothiazole derivatives stabilize the thermolabile p53 cancer mutant Y220C and show anticancer activity in p53-Y220C cell lines. Eur J Med Chem. 2018;152:101–114. 29702446 10.1016/j.ejmech.2018.04.035 PMC5986712

[60] Synnott NC, Madden SF, Bykov VJN, Crown J, Wiman KG, Duffy MJ. The mutant p53-targeting compound APR-246 induces ROS-modulating genes in breast cancer cells. Transl Oncol. 2018;11(6):1343–1349. 30196236 10.1016/j.tranon.2018.08.009 PMC6132178

[61] Puzio-Kuter AM, Xu L, McBrayer MK, Dominique R, Li HH, Fahr BJ, Brown AM, Wiebesiek AE, Russo BM, Mulligan CL, et al. Restoration of the tumor suppressor function of Y220C-mutant p53 by rezatapopt, a small-molecule reactivator. Cancer Discov. 2025;15(6):1159–1179. 39945593 10.1158/2159-8290.CD-24-1421 PMC12130801

[62] Vu BT, Dominique R, Fahr BJ, Li HH, Fry DC, Xu L, Yang H, Puzio-Kuter A, Good A, Liu B, et al. Discovery of rezatapopt (PC14586), a first-in-class, small-molecule reactivator of p53 Y220C mutant in development. ACS Med Chem Lett. 2025;16(1):34–39. 39811143 10.1021/acsmedchemlett.4c00379 PMC11726359

[63] Zhu J, Singh M, Selivanova G, Peuget S. Pifithrin-alpha alters p53 post-translational modifications pattern and differentially inhibits p53 target genes. Sci Rep. 2020;10(1):1049. 31974452 10.1038/s41598-020-58051-1 PMC6978515

[64] Liu X, Wilcken R, Joerger AC, Chuckowree IS, Amin J, Spencer J, Fersht AR. Small molecule induced reactivation of mutant p53 in cancer cells. Nucleic Acids Res. 2013;41(12):6034–6044. 23630318 10.1093/nar/gkt305 PMC3695503

[65] Huang F, Han X, Xiao X, Zhou J. Covalent warheads targeting cysteine residue: the promising approach in drug development. Molecules. 2022;27(22):7728. 36431829 10.3390/molecules27227728 PMC9694382

