# Supplementary material for: In vitro screening of compounds for targeting gastric cancer with Y220C p53 mutation: a molecule combining zinc chelation and a Michael acceptor drives CDKN1 and BBC3 expression to restore a p53-dependent cytotoxicity
Source: J Enzyme Inhib Med Chem. 2026 May 21;41(1):2638836. doi: 10.1080/14756366.2026.2638836 (PMC13195717; doi:10.1080/14756366.2026.2638836)
Supplement: Supplemetary materials VF-cl.docx [file IENZ_A_2638836_SM3788.docx]

**SUPPLEMENTARY MATERIALS AND METHODS**

**Table SM1: Sequence of oligonucleotide primers used for qPCR**

| **Targeted genes** | **Sequences of forward and reverse primers** |
| --- | --- |
| ***p53*** | Forward primer: 5’ CAG CCA AGT CTG TGA CTT GCA 3’  Reverse primer: 5’ GTG TGG AAT CAA CCC ACA GCT 3’ |
| ***p21*** | Forward primer: 5’ ATG AAA TTC ACC CCC TTT CC 3’  Reverse primer: 5’ CCC TAG GCT GCT CAC TTC 3’ |
| ***PUMA*** | Forward primer: 5’ GTA AGG CCA GGA GTC CCA TGA TCA 3’  Reverse primer: 5’ ACG ACC TCA ACC GCA CAG TAC GA 3’ |
| ***ZMAT3*** | Forward primer: 5’ GCC AGG AAA GAA GGG AAT G 3’  Reverse primer: 5’ GCG GGG ATT GAA GTA AGG AC 3’ |
| ***GAPDH*** | Forward primer: 5’ CAC TTT GTC AAG CTC 3’  Reverse primer: 5’ GGT CTA CAT GGC AAC TGT GA 3’ |
| ***RPLPO*** | Forward primer: 5’ CAA GGC TGT GGT GCT GAT GG 3’  Reverse primer: 5’ CCG GAT ATG AGG CAG CAG TT 3’ |

**RNA sequencing and *in silico* analysis**

RNA was extracted from AGS cells using TRIzol-mediated cell lysis. After extraction and RNA precipitation, supernatants were removed and the RNA pellet was washed with 75% EtOH, centrifuged at 9000 × *g* for 5 min at 4 °C, and again, 75% EtOH was added. Then, the RNA was resuspended in nuclease-free H_2_O and quantified using a NanoDrop Spectrophotometer (Thermo Scientific, Waltham, MA, USA). To identify the deregulated genes in the mouse microenvironment of the tumor, RNA-Seq was performed on extracted total RNAs and the sequences obtained were selectively aligned on the human genome (hg38) using STAR version 2.5.3a. The quantification step was performed using HTSeq-count version 0.6.1p1, with annotations from Ensembl version 103 (Homo sapiens) and then data were further processed with AltAnalyze version 2. After standard normalization, deregulated genes with log2 fold change >1.3 and adjusted *p*-value <0.05 were selected and pathways enrichment analyses were performed using multiple databases (e.g., DAVID, STRING, Reactome, TRAP, Biomarkers).

**Protein expression and purification**

A stabilized quadruple mutant variant (QM; M133L/V203A/N239D/N268D) of the human p53 DBD (residues 94-312) and its Y220C mutant were expressed and purified as described previously [1]. Briefly, we used a pET24a-based expression vector to encode a fusion protein containing: an N-terminal 6xHis tag, the lipoyl domain of the dihydrolipoamide acetyltransferase from *Bacillus stearothermophilus*, a TEV protease cleavage site, and the p53 DBD variant (residues 94−312) of interest. The recombinant proteins were expressed in *E. coli* C41. The expression process involved the following steps: (i) Cells containing the expression vector were grown in 2TY medium at 37 °C to an OD_600_ = 0.6−0.8, (ii) the temperature was then lowered to 20 °C, and protein expression was induced by adding IPTG (0.5 mM), (iii) 0.1 mM zinc chloride was added to the cells. Cells were grown overnight and then broken by sonication. The p53 DBD variants were purified using a nickel column, followed by TEV protease cleavage overnight, a second purification step on a heparin column, and a final gel filtration chromatography step using a Superdex 75 column. After purification, the p53 DBD mutants were concentrated to 6 mg mL−1, flash frozen in liquid nitrogen, and stored at -80 °C.

**Mass spectrometry**

The p53-Y220C QM DBD (20 µM) was incubated with AG compounds at a concentration of 500 µM either for 4 h at 4 °C or 16 h at room temperature prior to mass spectrometry measurement in MS buffer (25 mM HEPES, pH 7.5, 200 mM NaCl, and 0.5 mM TCEP, 0.04% formic acid). An Agilent 6230 electrospray ionization time-of-flight mass spectrometer was used. A sample volume of 5 μL was injected onto a guard column, followed by gradient elution (from water to acetonitrile supplemented with 0.1% formic acid) at a flow rate of 0.4 mL/min. Data acquisition was achieved using the MassHunter LC/MS data acquisition software, and subsequent analysis was performed with the BioConfirm vB.08.00 tool, both included in the Agilent Technology software suite (<https://www.agilent.com/en/product/software-informatics/mass-spectrometry-software>). The peak intensities of both non-alkylated and alkylated p53-DBD Y220C species were plotted.

**Differential scanning fluorimetry (DSF) measurements**

The effects of AG3 and analogues on the melting temperature of recombinant p53 QM DBD were determined by DSF in a 96-well plate (Starlab). The WT and Y220C QM DBDs (5 µM) were incubated with different concentrations of AG compounds at room temperature in buffer containing 25 mM HEPES, pH 7.5, 250 mM NaCl, and 0.5 mM TCEP (with a final DMSO concentration of 2.4% in all experiments and controls), prior to thermal shift assays. SYPRO Orange (5000×, Invitrogen) was added at a dilution of 1:1000 (final concentration of 5x). Protein unfolding profiles were recorded using an MX3005P real-time qPCR instrument (Agilent; excitation/emission filters = 492/610 nm) while increasing the temperature from 25 to 95 °C at a heating rate of 3 °C/min. *T*_m_ values were calculated after fitting the fluorescence curves to the Boltzmann equation, and the mean of triplicate measurements is given, with a standard deviation of ± 0.1 °C in all cases.

**GSH NMR studies: rate constant determination**

850 µL of 50 mM KPi buffer solution (pH 7 or 6.5), 20 µL of the appropriate pyrimidine stock solution (50 mM in DMSO-d6), 20 µL of TMSP stock solution (22.2 mM in DI water), 30 µL DMSO-d6, and 80 µL of GSH stock solution (0.125 M in DI water) were successively added/mixed at room temperature. 600 µL of this freshly prepared solution was poured into an NMR tube and immediately transferred to the 500 MHz NMR instrument for acquisition. ^1^H spectra (16 scans) were recorded every 60 min under water suppression conditions. Following acquisition, spectra were analyzed using ACDLabs.

### **Synthesis and MS**

### **1.1.1 General experimental for synthesis of AG1, AG2, AG3 and AG3-Red**

NMR data were obtained for ^1^H at 400 MHz or 500 MHz and ^13^C at 100 MHz or 125 MHz. Chemical shifts were recorded at ambient temperature (300 K) on a Bruker AVA500 or PRO500 spectrometer. The chemical shifts were reported in ppm and the residual solvent peaks (CDCl_3_ and *d*^6^-DMSO) were used as references. Chemical shifts (δ values) are reported in parts-per-million (ppm). ^1^H NMR data is reported as chemical shift, multiplicity (*s* = singlet, *d* = doublet, *t* = triplet, *q* = quartet, *m* = multiplet), coupling constants (*J* = value, Hz), relative intensity, and interpretation. ^13^C NMR data is reported as a chemical shift. Commercial reagents include 1,10-phenanthroline-5-amine (Fluorochem, product number: 235199) and 4'-chloro-2,2':6',2''-terpyridine (Fluorochem, product number: 329568). Anhydrous solvents were obtained from retail suppliers or a Glass Contour solvent purification system (SPS). MS was obtained by electrospray ionisation (ESI) on a Waters Synapt G2 or Bruker microTOF II. Mass-to-charge ratios (*m/z*) of all molecular ions ([M]^+/-^), their intensities, and fragment or adductions and their intensity are reported. The CARY UV/Vis spectrometer obtained UV-vis results from CARY 300 system.

Compounds AG1 [2], AG2 [3] and AG3 [4] were synthesised according to literature procedures. For clarity the methods have been reproduced below.

### **1.1.2 Synthesis of AG1:**

### **1.1.2.1 Di(2-pyridyl) methylamine, [5] (1) {Unjaroen, 2017 #69}{Unjaroen, 2017 #69}{Unjaroen, 2017 #69}{Unjaroen, 2017 #69}**

Zinc powder (3.12 g, 47.76 mmol) was added slowly at room temperature to a mixture of ammonium acetate (1.02 g, 13.26 mmol) and di(pyridine-2-yl) methanone oxime [6] (2.64 g, 13.26 mmol) in 25% NH_3_/H_2_O (20 mL) and ethanol (15 mL). The mixture was heated at reflux for 2.5 h. After stirring at room temperature overnight, the solution was filtered over Celite and washed with ethanol. After evaporating the solvent in vacuo, 2 M NaOH (30 mL) was added to the residue, and the product was extracted with CH_2_Cl_2_ (3×30 mL). The combined CH_2_Cl_2_ layers were washed with brine (20 mL), dried (over anhydrous Na_2_SO_4_), and removed from the solvent in a vacuum. Crude light-yellow oil, 2.0 g, 83% yield. ^1^H NMR (500 MHz, CDCl_3_) δ = 8.54 (dq, *J* = 5.0, 1.0, 0.9 Hz, 2H), 7.61 (td, *J* = 7.8, 1.8 Hz, 2H), 7.37 (d, *J* = 7.8 Hz, 2H), 7.12 (ddd, *J* = 7.6, 4.9, 1.2 Hz, 2H), 5.31 (s, 1H), 2.41 (s, 2H). ^13^C NMR (125 MHz, CDCl_3_) δ = 162.9, 149.3, 136.7, 122.2, 121.9, 62.5. The data were in accordance with literature values.

### **1.1.2.2 AG1**

A solution of di(2-pyridyl) methylamine (2.0 g, 10.8 mmol, 1 eq) in dry toluene (4 mL) was added dropwise to a stirred solution of maleic anhydride (1.1 g, 10.8 mmol, 1 eq) in dry toluene (20 mL) at room temperature. After the addition, the resulting suspension was stirred for 1 h, and then ZnCl_2_ (1.5 g, 10.8 mmol, 1 eq) was added in one portion. While the resulting reaction mixture was heated (80 °C), a solution of HMDS (([Bis(trimethylsilyl)amine](https://www.bing.com/ck/a?!&&p=642404fe24cf873eJmltdHM9MTcwMDE3OTIwMCZpZ3VpZD0xMzE4OTQ0OC0xNjIwLTYwMmQtMzkyMC04NDllMTcxMDYxNjkmaW5zaWQ9NTIyNw&ptn=3&ver=2&hsh=3&fclid=13189448-1620-602d-3920-849e17106169&psq=HMDS+chemical+full+name&u=a1aHR0cHM6Ly9lbi53aWtpcGVkaWEub3JnL3dpa2kvQmlzKHRyaW1ldGh5bHNpbHlsKWFtaW5l&ntb=1)), 3.3 mL, 16.0 mmol, 1.5 eq) in dry toluene (4 mL) was added slowly over 10 min, and then the mixture was heated to reflux. After completion, the reaction mixture was cooled to room temperature and poured into saturated NH_4_Cl. The aqueous phase was extracted with ethyl acetate. The organic extract was washed with saturated brine and dried over anhydrous Na_2_SO_4_. The solution was concentrated under reduced pressure and the residue purified by silica filtration (silica gel, petroleum ether/EtOAc 4:1) to afford the desired compound as a light yellow solid, 760 mg, 26.5% yield. ^1^H NMR (400 MHz, *d*^6^-DMSO) δ = 8.54 (ddd, *J* = 4.8, 1.9, 1.0 Hz, 2H), 7.79 (td, *J* = 7.8, 1.9 Hz, 2H), 7.38 – 7.30 (m, 2H), 7.22 (dq, *J* = 7.8, 1.0 Hz, 2H), 7.11 (s, 2H), 6.49 (s, 1H). ^13^C NMR (100 MHz, *d*^6^-DMSO) δ = 170.7, 156.3, 148.9, 136.7, 134.8, 123.1, 122.8, 60.0. ESI HRMS: calcd. for C_15_H_11_N_3_O_2_+H^+^ 266.0924, found 266.0925. The data were in accordance with literature values.

### **1.1.3 Synthesis of AG2**

### **1.1.3.1 Synthesis of 2-(2,2′:6′,2″-terpyridine-4′-yloxy)ethylamine, [7] (2)**{Zhang, 2014 #77}

A suspension containing 930 mg (17.5 mmol, 5 eq) of powdered KOH in 10 mL of DMSO was prepared. To this mixture, 938 mg (3.5 mmol, 1 eq) of 4′-chloro-2, 2′: 6′,2″-terpyridine and 340 μL (3.9 mmol, 1.1 eq) of ethanolamine were added. The reaction mixture was heated to 40 °C for 2 h, followed by the addition of 40 mL of DCM. The organic phase was then washed with H_2_O (3×100 mL) and dried over MgSO_4_. After removing the solvent, the resulting product **(4)** (800 mg, 78%) was obtained as a light yellow solid and used into next step subsequently without additional purification.

### **1.1.3.2 Synthesis of *N*-[2-([2,2′6′,2″-terpyridin]-4′-yloxy)ethyl]maleimide, AG2 {Zhang, 2014 #77}**

A suspension of **2** (800 mg, 2.7 mmol) and maleic anhydride (1.3 g, 13.6 mmol) in CH_2_Cl_2_ (40 mL) was heated to reflux overnight. After cooling to room temperature, the resulting pale white solid was filtered, dried under vacuum, and then added to a suspension of NaOAc (1.3 g, 16 mmol) in Ac_2_O (40 mL) and heated overnight at 100 ^o^C. Upon completion, the reaction mixture was quenched by pouring into ice water (200 mL) until Ac_2_O was decomposed. After extraction with DCM (3 × 50 mL), the organic phase was collected, and excess solvent was evaporated. The crude solid was recrystallized from acetone, yielding a pale yellow solid (520 mg, 52%). ^1^H NMR (500 MHz, *d*^6^-DMSO) δ = 8.71 (ddd, *J* = 4.8, 1.8, 0.9 Hz, 2H), 8.59 (d, *J* = 8.0 Hz, 2H), 7.99 (td, *J* = 7.7, 1.8 Hz, 2H), 7.91 (s, 2H), 7.49 (ddd, *J* = 7.5, 4.8, 1.2 Hz, 2H), 7.05 (s, 2H), 4.39 (t, *J* = 5.5 Hz, 1H), 3.89 (t, *J* = 5.5 Hz, 1H). ^13^C NMR (125 MHz, *d*^6^-DMSO) δ = 170.8, 166.1, 156.7, 154.7, 149.2, 137.3, 134.6, 124.5, 120.8, 106.6, 65.0, 36.5. ESI HRMS: calcd. for C_21_H_16_N_4_O_3_+H^+^ 373.1296, found 373.1302. The data were in accordance with literature values.

### **1.1.4 Synthesis of AG3 and AG3-Red**

### **1.1.4.1 5-Maleimido-1,10-phenanthroline**, **AG3**

A suspension of 1,10-phenanthroline-5-amine (1.6 g, 8.2 mmol) and maleic anhydride (4.00 g, 40.8 mmol) in CH_2_Cl_2_ (80 mL) was heated to reflux overnight. After cooling to room temperature, the solid was filtered and washed with CH_2_Cl_2_. Then the solid (1.4 g, 5.2 mmol) was added to a suspension of NaOAc (8.0 g) in Ac_2_O (80 mL) and heated overnight at 100 ^o^C. After cooling to room temperature, the mixture was poured into ice water (200 mL) and stirred until the Ac_2_O was decomposed completely. After extraction with CH_2_Cl_2_ (3×150 mL), the organic phase was washed with H_2_O (3×150 mL), dried over MgSO_4_, and concentrated. The addition of hexane (100 mL) provoked precipitation of the product, which was then filtrated and recrystallised from acetone and hexane to give a light-yellow solid, 460 mg, 20% yield. ^1^H NMR (500 MHz, DMSO) δ 9.18 (ddd, *J* = 8.4, 4.3, 1.7 Hz, 2H), 8.54 (dd, *J* = 8.1, 1.8 Hz, 1H), 8.33 (dd, *J* = 8.3, 1.7 Hz, 1H), 8.10 (s, 1H), 7.84 (dd, *J* = 8.1, 4.3 Hz, 1H), 7.79 (dd, *J* = 8.3, 4.2 Hz, 1H), 7.35 (s, 2H). ^13^C NMR (125 MHz, DMSO) δ 170.4, 151.1, 150.5, 145.9, 145.4, 136.6, 135.2, 132.1, 127.9, 127.3, 126.6, 126.1, 123.9, 123.6. ESI HRMS: calcd. for C_16_H_9_N_3_O_2_+H^+^ 276.0767, found 276.0763. The data were in accordance with literature values.

**1.1.4.2 AG3 reduction**

|  | Mw | eq | mmol | mg | µ | ρ |
| --- | --- | --- | --- | --- | --- | --- |
| AG3 | 275.26 | 1.0 | 0.18 | 50 | - | - |

**Fig SM 1**: AG3 (50 mg, 1.0 eq) was dissolved in MeOH (3.0 ml) and loaded into the H-Cube® Mini Plus, equipped with a 30 mm CatCart® (10% Pd/C) at a system temperature of 30 ^o^C. The system was run in continuous flow mode at a rate of 1 ml per min for 90 min.

After 90 min resulting material was collected and concentrated to dryness yielding a colourless oil. This oil was triturated in Et_2_O using a sonicator for 15 min yielding an off-white solid which was collected by filtration and air dried leading to the isolation of 1-(1,10-phenanthrolin-5-yl)pyrrolidine-2,5-dione (25 mg, 50%), 97 % purity by LCMS. ^1^H NMR (600 MHz, DMSO-*d*_6_) δ 9.17 (dd, *J* = 9.2, 2.6 Hz, 2H), 8.57 (d, *J* = 8.2 Hz, 1H), 8.43 (d, *J* = 8.3 Hz, 1H), 8.00 (s, 1H), 7.84 (dd, *J* = 8.0, 4.3 Hz, 1H), 7.80 (dd, *J* = 8.3, 4.2 Hz, 1H), 3.11 – 3.01 (m, 2H), 2.97 – 2.88 (m, 2H). ^13^C NMR (151 MHz, DMSO-*d*_6_) δ 178.0, 151.5, 150.9, 146.2, 145.8, 137.1, 132.6, 128.5, 127.8, 127.7, 126.0, 124.3, 123.9, 29.6. [M+H] Predicted mass = 278.0930. Calculated mass = 278.0938.

## **1.1.5 Spectra of compounds (^1^H-NMR, ^13^C-NMR, and ESI-HRMS)**

**Fig. SM2: 500 MHz ^1^H-NMR spectrum of compound (1)**

**Fig. SM3. 125 MHz ^13^C-NMR spectrum of compound (1)**

**Fig. SM4. 400 MHz ^1^H-NMR spectrum of AG1**

**Fig. SM5. 100 MHz ^13^C-NMR spectrum of AG1**


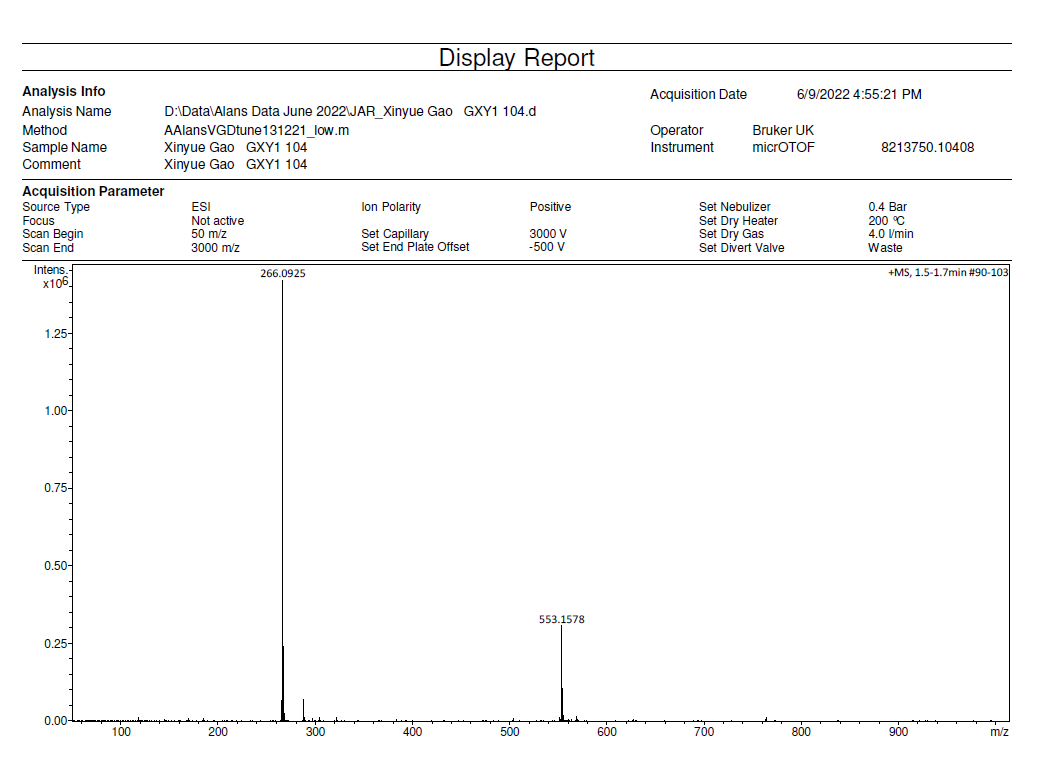

**Fig. SM6. EI-MS spectrum of AG1**


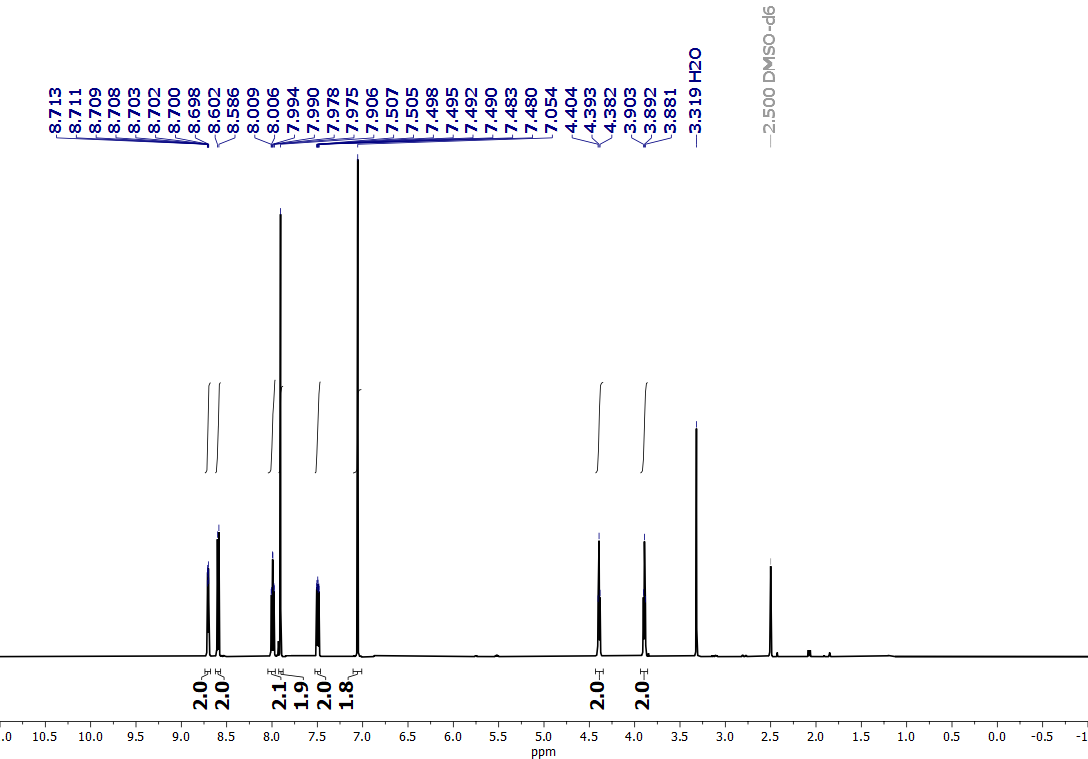

**Fig. SM7. 500 MHz ^1^H-NMR spectrum of compound AG2**

**Fig. SM8. 125 MHz ^13^C-NMR spectrum of compound AG2**


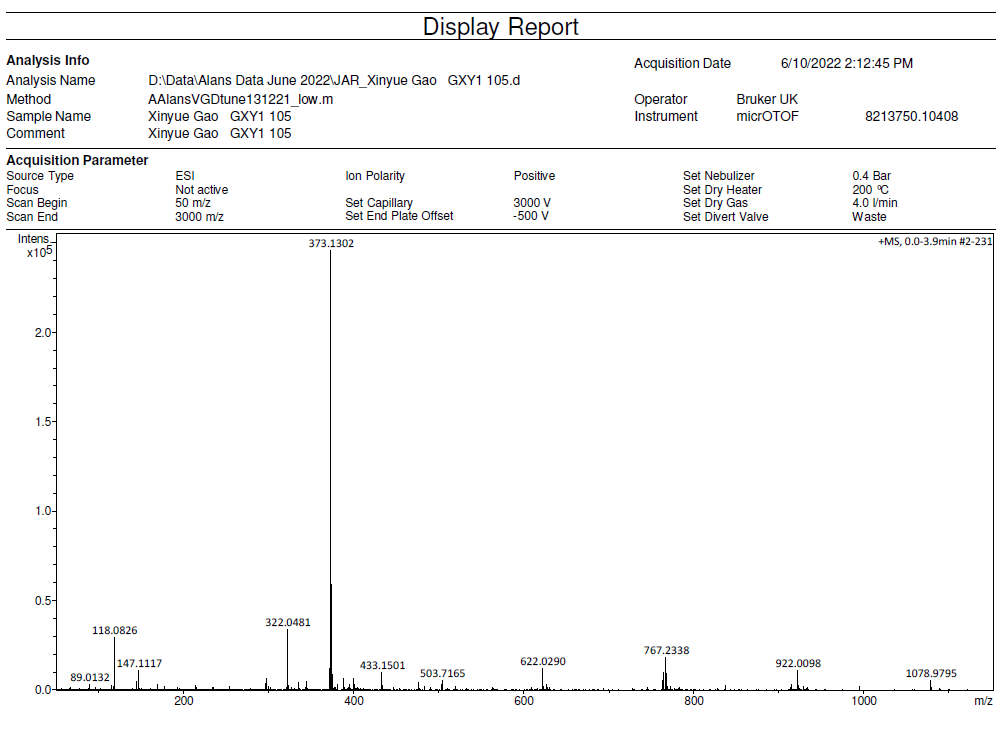

**Fig. SM9. EI-MS spectrum of compound AG2**

**Fig. SM10. 500 MHz ^1^H-NMR spectrum of compound AG3**

**Fig. SM11. 125 MHz ^13^C-NMR spectrum of compound AG3**

**Fig. SM12. EI-MS spectrum of compound AG3**

**1.1.6 Zinc binding studies on AG3:**

**1.1.6.1** NMR study of zinc binding in DMSO

To a solution of AG3 in d_6_-DMSO (1 mM) was added a solution of ZnCl_2_ in D_2_O (2.2 μL, 113 mM, 0.5 eq.) and the NMR spectrum recorded. Further portions of ZnCl_2_ solution up to 10 eq. were added. For spectra see Fig. 7.

**1.1.6.2** NMR study of zinc binding in DMSO:D_2_O 1:3

Initially, an NMR spectrum of AG3 in d_6_-DMSO:D_2_O 1:3 (1 mM, pH 7.6) was recorded. To this solution was added a solution of ZnCl_2_ in D_2_O (4.4 μL, 113 mM, 1 eq.) and the NMR spectrum recorded. Up to 2 eq. was added. After leaving at rt for 4 weeks ring, opening was complete (see Figure SM9 a and b).

**References**

[1] D.I. Balourdas, A.M. Markl, A. Kramer, G. Settanni, A.C. Joerger, Structural basis of p53 inactivation by cavity-creating cancer mutations and its implications for the development of mutant p53 reactivators, Cell death & disease, 15 (2024) 408.

[2] N. Madern, N. Queyriaux, A. Chevalley, M. Ghasemi, O. Nicolotti, I. Ciofini, G.F. Mangiatordi, M. Salmain, Piano-stool d-rhodium(III) complexes of chelating pyridine-based ligands and their papain bioconjugates for the catalysis of transfer hydrogenation of aryl ketones in aqueous medium, J Mol Catal B-Enzym, 122 (2015) 314-322.

[3] C. Zhang, P. Srivastava, K. Ellis-Guardiola, J.C. Lewis, Manganese terpyridine artificial metalloenzymes for benzylic oxygenation and olefin epoxidation, Tetrahedron, 70 (2014) 4245-4249.

[4] S.A. Trammell, H.M. Goldston, Jr., P.T. Tran, L.M. Tender, D.W. Conrad, D.E. Benson, H.W. Hellinga, Synthesis and characterization of a ruthenium(II)-based redox conjugate for reagentless biosensing, Bioconjugate chemistry, 12 (2001) 643-647.

[5] D. Unjaroen, M. Swart, W.R. Browne, Electrochemical Polymerization of Iron(III) Polypyridyl Complexes through C-C Coupling of Redox Non-innocent Phenolato Ligands, Inorganic chemistry, 56 (2017) 470-479.

[6] J. Chang, S. Plummer, E.S. Berman, D. Striplin, D. Blauch, Synthesis and characterization of bis(di-2-pyridylmethanamine)ruthenium(II), Inorganic chemistry, 43 (2004) 1735-1742.

[7] P.R. Andres, R. Lunkwitz, G.R. Pabst, K. Böhn, D. Wouters, S. Schmatloch, U.S. Schubert, New 4′-functionalized 2,2′:6′,2"-terpyridines for applications in macromolecular chemistry and nanoscience, Eur J Org Chem, 2003 (2003) 3769-3776.
